# Mycotoxin contamination in food and feed: Occurrence, co-occurrence, combined impacts on human and animal health, climate change implications, risk assessment, and mitigation strategies

**DOI:** 10.1007/s12550-026-00679-5

**Published:** 2026-09-15

**Authors:** Oluwasola Abayomi Adelusi, Viola Onyinye Okechukwu, Julianah Olayemi Odukoya, Rumbidzai Changwa, Adeola Oluwakemi Aasa, Oluwaseun Mary Oladeji, Nnamdi Nwulu, Kulsum Kondiah, Oluwafemi Ayodeji Adebo, Adewale Olusegun Obadina, Hany S. El-Mesery, Sarah De Saeger, Patrick Berka Njobeh

**Affiliations:** 1 Department of Biotechnology and Food Technology, Faculty of Science, University of Johannesburg, Doornfontein Campus, P.O. Box 17011, Doornfontein, Gauteng 2028 South Africa; 2 Department of Biochemistry and Microbiology, Faculty of Natural and Agricultural Sciences, University of Pretoria, Pretoria, South Africa; 3 Department of Biology and Environmental Science, Faculty of Science, Sefako Makgatho Health Sciences University, P.O. Box 139, Pretoria, 0204 South Africa; 4 Department of Electrical and Electronic Engineering, University of Johannesburg, Johannesburg, 2006 South Africa; 5 Centre for Innovative Food Research (CIFR), Department of Biotechnology and Food Technology, University of Johannesburg, Doornfontein Campus, P.O. Box 17011, Johannesburg, 2028 South Africa; 6 Department of Food Science and Technology, Federal University of Agriculture, Abeokuta, Nigeria; 7 School of Energy and Power Engineering, Jiangsu University, Zhenjiang, 212013 China; 8 Centre of Excellence in Mycotoxicology and Public Health (CEMPH), Faculty of Pharmaceutical Sciences, Universiteit Gent, Ghent, 9000 Belgium

**Keywords:** Fungal toxins, Agricultural products, Public health risks, Regulatory standards, Control measures

## Abstract

Mycotoxins are toxic secondary metabolites produced by numerous fungi that infect crops during pre- and post-harvest stages. Aflatoxins, ochratoxins, fumonisins, zearalenone, and trichothecenes are of greatest concern due to their widespread occurrence and high toxicity. Consumption of mycotoxin-contaminated food and feed poses serious public health risks, as these compounds are linked to carcinogenic, neurotoxic, nephrotoxic, hepatotoxic, and immunotoxic effects. The frequent co-occurrence of multiple mycotoxins further amplifies health risks through additive or synergistic interactions and contributes to substantial economic losses. This review provides an updated overview of the global distribution and co-occurrence of major mycotoxins in agricultural products, their individual and combined toxicological effects on human and animal health, and the regulatory standards and maximum permitted levels for mycotoxins in food and feed. In addition, this review discusses the impact of climate change on mycotoxin contamination in food and feed, exposure, and associated health risks. It further outlines mycotoxin risk assessment procedures and highlights mitigation strategies for reducing contamination and limiting human and animal exposure risks. Ultimately, this review underscores the urgent need for integrated mycotoxin risk management strategies and strengthened global surveillance systems to safeguard food safety, protect human and animal health, and enhance food security.

**Supplementary Information:**

The online version contains supplementary material available at https://doi.org/10.1007/s12550-026-00679-5.

## Highlights

Recent information on the occurrence and co-occurrence of mycotoxins in major agricultural commodities is synthesized.

The individual and combined health effects of mycotoxins in humans and animals are critically examined.

The influence of climate change on mycotoxin contamination patterns and exposure risks is evaluated.

Current approaches to mycotoxin risk assessment in food and feed are highlighted.

Mitigation strategies aimed at reducing human and animal exposure to mycotoxins are discussed.

**Graphical abstract:**

**Supplementary Information:**

The online version contains supplementary material available at https://doi.org/10.1007/s12550-026-00679-5.

## Graphical abstract

**Figure:**
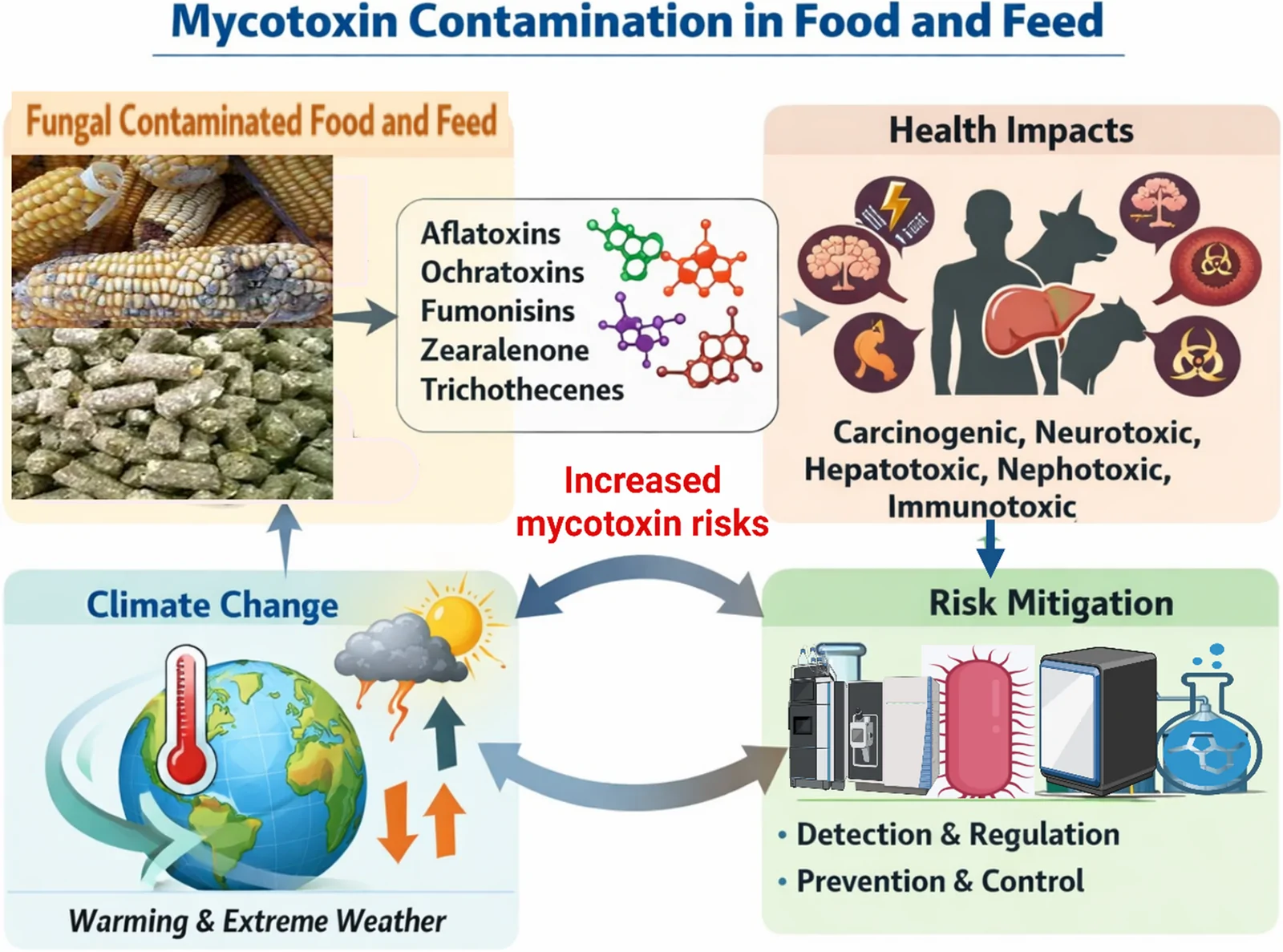

## Introduction

Mycotoxins are a class of compounds primarily produced via secondary metabolism in certain filamentous fungi. They are considered primary food and feed contaminants owing to their detrimental impact on human and animal health (Yan et al. 2026). The proliferation of toxigenic fungal species and subsequent mycotoxin formation in food and feedstuff occur under suitable eco-physiological conditions of water activity, relative humidity, temperature, and pH throughout the entire agricultural food production chain, that is, in the field, during harvest, storage, and in transit (Platzer et al. 2025; Yan et al. 2026). Climate change also represents another major agro-ecosystem driving force for mycotoxin production in agricultural products (Medina et al. 2017; Yan et al. 2026). Currently, more than 400 mycotoxins have been characterized, but only a limited number have been extensively investigated due to their toxicological effects on humans and animals (Chen et al. 2020). The most prevalent mycotoxins with the highest public health and agro-economic importance are ochratoxins (OTs), aflatoxins (AFs), zearalenone (ZEN), fumonisins (FBs), and trichothecenes (TCTs), including nivalenol (NIV), deoxynivalenol (DON), T-2 and HT-2 toxins (Smith et al. 2016; Akinmoladun et al. 2025). It is worth noting that the simultaneous presence of multiple mycotoxins in agricultural products is common, as numerous fungal species can produce more than one variety of mycotoxins. Most mycotoxins are heat-stable and are not completely degraded in human and animal diets by conventional processing. However, certain food processes, including sorting, blanching, milling, nixtamalization, and extrusion, can drastically lower contamination levels depending on the mycotoxin type, processing conditions, and food matrix (Tibola et al. 2016; Adelusi et al. 2026).

The health risks associated with mycotoxins are determined not only by contamination and degradation during food processing but also by their degree of bioaccessibility after consumption. Bioaccessibility refers to the percentage of mycotoxins released from the food matrix during digestion and available for absorption in the gut (Gordon et al. 2026; Massarolo et al. 2025). Monteiro et al. (2026) confirmed that certain factors, including food composition, digestion conditions, and interactions with other food components, can all influence the release of mycotoxins, thereby increasing or decreasing their availability for absorption. As a result, the concentration of mycotoxins in food cannot be used as the primary indicator of internal exposure in humans or animals. Considering bioavailability in the risk assessment of mycotoxins provides a more realistic approach to dietary exposure evaluation, as such information is imperative to inform risk management and support effective control measures (Bogueva and McClements 2023).

Human exposure to mycotoxins occurs directly via the consumption of contaminated plant-based foods and animal products like eggs, meat, and milk that contain mycotoxin residues. Another route of exposure is inhalation, particularly in an industrial environment where toxigenic fungal spp. develop on materials and products (Marcelloni et al. 2024). Mycotoxin exposure is more prevalent in regions that lack effective pest control methods for crops, transportation systems, and storage facilities (Omotayo et al. 2019a; Bereka et al. 2022), as well as in regions experiencing significant economic hardship, wherein individuals are more concerned about what to eat (availability) and not safety (Gbashi et al. 2018). Regular exposure to mycotoxins is also widespread in areas lacking regulations and laws aimed at safeguarding the food consumption of the populace. Also, in developing nations where mishandling of food is rampant, there is a possibility of a higher risk of mycotoxin exposure (Dey et al. 2022), thus rendering the population susceptible to acute or chronic mycotoxin-induced diseases. Other key factors contributing to the presence of mycotoxins in food and feed are climate and environmental conditions, as well as geographical differences (Adelusi et al. 2026; Gbashi et al. 2024).

According to Adelusi et al. (2022), mycotoxins may exert carcinogenic, mutagenic, teratogenic, neurotoxic, estrogenic, and immunosuppressive consequences on humans and animals. Pitt and Miller (2017) reported that mycotoxins have been linked to epidemics in humans and animals over the past three decades, leading to severe adverse health effects and contributing to substantial public health and economic burdens globally. Moreover, these contaminants were also associated with the onset of alimentary toxic aleukia (ATA), a chronic disease that claimed the lives of more than one hundred thousand Russians between 1942 and 1948 (Tripathi and Alam 2020). The deaths of 125 individuals in Kenya in 2004 and 16 people in Tanzania in 2016 after consuming AF-contaminated foods are a good illustration (Kamala et al. 2018). Furthermore, dietary exposure to mycotoxins, particularly AF, has been linked to malnutrition and growth impairment in children from Togo and Benin (Gong et al. 2002), Kenya (Okoth and Ohingo 2004; Ankwasa et al. 2021), Tanzania (Chen et al. 2018), and Malawi (Matchado et al. 2023). Furthermore, mycotoxins also cause several detrimental effects, such as reduced feed intake, impaired nutrient absorption, weight loss, and compromised immune function in animals (Rehman et al. 2022; Akinmoladun et al. 2025). As reported by da Rocha et al. (2014), ingestion of AF-contaminated feed by turkeys, pigs, and chickens severely suppressed immune response, which can result in a substantial financial burden. In addition to their well-documented carcinogenic, immunotoxic, nephrotoxic, and hepatotoxic effects, mycotoxins are also emerging as neurodegenerative agents. Chronic exposure to these harmful substances can cause oxidative stress, mitochondrial damage, disruption of the blood-brain barrier, and nervous system inflammation, all of which lead to cell death via apoptosis and synaptic dysfunction, as seen in Alzheimer’s and Parkinson’s patients (Nie et al. 2025; Ramos et al. 2025).

More importantly, the co-occurrence of mycotoxins in food and feed can result in additive, synergistic, or antagonistic interactions. While additive and synergistic interactions increase overall toxicity, antagonistic interactions may lessen the toxic response (Smith et al. 2016; Ren et al. 2017; Changwa et al. 2021; Mandal et al. 2024; Pavlov et al. 2025). Such unpredictability of interactions complicates human health risk assessment since the effects of mycotoxin combinations cannot be predicted from individual toxins alone. As a result of their proven health hazards in humans, the International Agency for Research on Cancer (IARC) categorizes certain mycotoxins based on their carcinogenic potential. For instance, Aflatoxin B_1_ (AFB_1_) is classified in Group 1 (carcinogen), FB_1_ and OTA in Group 2B (possible carcinogens), while ZEN and DON are classified as Group 3 (not classifiable as to their carcinogenicity to humans due to inadequate evidence in experimental animals and humans) [IARC, 1993]. In addition to IARC classifications, other regulatory bodies, such as the European Food Safety Authority (EFSA) and the U.S. Food and Drug Administration (FDA), have set maximum allowable limits for mycotoxins in food and feed products. These regulations are designed to protect public health and ensure food safety. It is crucial to mention that the adverse effects of mycotoxins in humans and animals are influenced by several factors, such as gender, body weight, age, as well as the type and amount of toxins to which they are exposed (Zain 2011).

Besides their impacts on human and animal health, the economic effects associated with mycotoxin contamination can be significant. They can affect food producers in different ways, ranging from losses in crop production, costs of testing and monitoring the toxins in food and feed, trade barriers, as well as food spoilage (Gbashi et al. 2018). According to estimates by the Food and Agriculture Organization (FAO) of the United Nations, approximately 25% of global food and feed production is contaminated with mycotoxins (Duarte et al. 2010). However, recent surveillance studies using advanced analytical methods have reported substantially higher contamination rates, often exceeding 60–80% depending on the commodity and geographical region (Eskola et al. 2020; BIOMIN, 2020; Budagova et al. 2025). Furthermore, mycotoxin contamination usually renders commodities less competitive in the export market. As reviewed by Gbashi et al. (2018), mycotoxin contamination remains a leading cause of agricultural commodity rejections at European borders, as reported by the EU Rapid Alert System for Food and Feed (RASFF). This highlights the critical role of mycotoxin risk assessment in identifying, evaluating, and managing food and feed safety risks within regulatory frameworks.

Mycotoxin risk assessment is a key process for evaluating potential health hazards associated with naturally occurring toxins in food and feed, integrating hazard identification, hazard characterization, exposure assessment, and risk characterization. Reliable assessment depends on robust analytical methods, including chromatographic techniques such as high-performance liquid chromatography (HPLC), liquid chromatography–tandem mass spectrometry (LC–MS/MS), ultra-high-performance liquid chromatography–mass spectrometry (UHPLC-MS), and gas chromatography–mass spectrometry (GC–MS), as well as immunochemical assays (Okechukwu et al. 2023). Recent advances, including high-resolution mass spectrometry, biosensors, and spectroscopic tools, have further enhanced the detection and surveillance of both regulated and emerging mycotoxins across diverse food and feed matrices (Okechukwu et al. 2023). In recent years, numerous studies have reviewed the occurrence in food and feed (Rai et al. 2020; Akinmoladun et al. 2025; Kovač and Jurčević, 2025), their detection techniques (Iqbal 2021; Okechukwu et al. 2023), and strategies aimed at regulating exposure (Meneely et al. 2023; Wang et al. 2025). Furthermore, attention has also been directed towards the detrimental impacts of mycotoxins on human and animal well-being (Wangia-Dixon et al., 2020; Rai et al. 2020; Peivasteh-Roudsari et al. 2022).

Despite valuable insights from existing reviews, limited attention has been given to mycotoxin co-occurrence, their combined health effects in humans and animals, climate change–driven contamination, and, critically, mycotoxin risk assessment. This limitation is imperative as humans and animals are frequently exposed to multiple mycotoxins simultaneously, which can increase toxicity, while climate change continues to intensify fungal growth and toxin production. Therefore, this review addresses these gaps by integrating mycotoxin co-occurrence and interactive toxic effects into health risk assessment, while explicitly linking climate change, contamination dynamics, exposure pathways, and risk assessment within a unified framework. This approach will help to identify emerging risks and critical research gaps to support improved food safety risk assessment and mitigation strategies. By placing mycotoxin risk assessment at the core, the review provides a climate-smart, risk-based perspective that supports evidence-based mitigation, informed regulatory decision-making, and enhanced protection of human and animal health.

## Significant foodborne mycotoxins

### Aflatoxins

Aflatoxins are a group of toxic metabolites primarily produced by *Aspergillus* spp., including *A. flavus*, *A. parasiticus*, *A. ochraceoroseus*, *A. minisclerotigenes*, and *A. nomius* (Varga et al. 2009). The main types of AFs are AFB_1_, AFB_2_, AFG_1_, and AFG_2_, where the letters B and G denote blue and green fluorescence under ultraviolet light, and the numbers 1 and 2 indicate differences in chromatographic mobility (relative migration) in thin-layer chromatography. AF occupies a central role in mycotoxin analysis, with both B- and G-types primarily found in nuts, grains, and their by-products, including animal feeds and feed ingredients, whereas their biotransformation products, AFM_1_ and AFM_2_, are frequently found in milk and milk products. Among all AFs, AFB_1_ is indeed considered the most potent and toxic. AF prevalence in food and feed has been reported globally. Njoroge et al. (2017) recorded high AFB_1_ contamination levels of 11,000 and 3,000 µg/kg in groundnut kernel and milled groundnut powder from Zambia. The authors suggested that the elevated AF levels found in the groundnut products might be attributed to the vendors’ failure to sort out poor-quality kernels from the batch prior to the milling process. These elevated AF levels pose an escalated health risk to both humans and animals that consume such contaminated groundnut products. Another study reported total AF contamination in freshly harvested maize in rural Kenya at levels up to 411 µg/kg (Nabwire et al. 2020). Ng’ambi et al. (2022) investigated 1294 maize samples collected across four maize-producing districts in Malawi, namely Rumphi, Nsanje, Mzuzu, and Salima, for total AFs. They found that above 75% of the maize samples were contaminated with total AFs. The mean total AF levels in the maize samples were 8.5 ± 2.2, 100 ± 23.1, 11.9 ± 3.8, and 13.3 ± 1.7, respectively, surpassing the EU’s regulatory limit of 4 µg/kg. Additionally, the study’s results indicate that the contamination of maize with AF in the areas is driven by climate factors.

In the case of animal feed, AF levels in fish feed were also examined in several Kenyan sub-counties, with the highest amount coming from Kieni West County at a range of 1.76–39.7 µg/kg (Mwihia et al. 2018). Fish farmers from various regions in the country have reported fish with poor growth rates, loss of appetite, tumours, and high mortality rates. The AF-contaminated fish feed may be responsible for these undesirable traits (Mwihia et al. 2018). AF contamination of dairy cattle feeds from smallholder dairy farming systems in the South African provinces of Limpopo and the Free State was also reported by Changwa et al. (2021), with the highest AFB_1_ and AFG_1_ of 30.2 and 23.1 µg/kg, respectively. Likewise, Xiong et al. (2018) reported the occurrence of AFB_1_ in 174 feedstuffs (maize, soybean, peanut, wheat bran, and cottonseed meal) destined for Chinese dairy cattle. Their result showed that 61 (35.1%) of the feed samples contained AFB_1_, with a mean level of 24.4 µg/kg, above the permissible limits of 5 µg/kg set by the EU for dairy cattle feed ingredients.

According to Okechukwu et al. (2024), AFs, particularly AFB_1_, exert severe toxic effects primarily through metabolic activation in the liver, where AFB_1_ is converted to the highly reactive AFB_1_−8,9-epoxide (AFBO). This metabolite binds to DNA, especially at the N-7 guanine position, leading to genetic damage and carcinogenesis. AFB_1_ is among the most potent naturally occurring hepatotoxic and hepatocarcinogenic mycotoxins, posing a high risk of liver cancer, especially in individuals co-infected with hepatitis B virus (HBV), where strong synergistic effects have been reported. In sub-Saharan Africa, the high consumption of AFs and prevalence of HBV contribute to approximately 250,000 deaths annually from hepatocellular carcinoma (Zain 2011). Ali (2019) affirmed that children with chronic HBV infection exhibit higher levels of AFB_1_–DNA adducts, indicating increased susceptibility to AF-related toxicity. More so, AF can disrupt intestinal microbiota, potentially leading to metabolic disorders (Akinrinmade et al. 2016) and contribute to protein deficiency diseases like kwashiorkor, which can be fatal in children (Freire and da Rocha, 2017). Similarly, AFs in animal feed can negatively impact animal performance. Ruminants consuming AF-contaminated feed may show altered plasma metabolites, indicating potential health issues. For example, dairy cattle fed 100 µg/kg of AFB_1_ exhibited decreased enzyme concentrations (Sulzberger et al. 2017). AFB_1_ can also affect bovine embryos and reduce bull sperm viability (Komsky-Elbaz et al. 2018; Jiang et al. 2019). In South Africa, two major outbreaks of canine aflatoxicosis linked to aflatoxin-contaminated dog food have been reported. The first occurred in 1987 and resulted in ten dog deaths with severe hepatic lesions, including fibroplasia, bile duct proliferation, and necrosis (Bastianello et al. 1987). A second outbreak in Gauteng in 2011 caused over 220 dog deaths, with many others severely affected, due to aflatoxin-contaminated feed (Arnot et al. 2012). Table 1 summarizes the adverse human health consequences of AFs and other important mycotoxins such as OTA, FBs, ZEN, and TCTs, including their neurodegenerative properties.

**Table 1:** Major mycotoxins, producing fungal species, and their effects on human health (IARC, 1993; 2012; Omotayo et al. 2019b; Houbraken et al. 2020; El-Sayed et al. 2022; Nie et al. 2025; Ramos et al. 2025)

Mycotoxins	Fungal species	IARC classification	Effects on human health
Aflatoxins (AFB_1_, AFB_2_, AFG_1_, and AFG_2_)	*A. flavus*,* A. parasiticus*,* A. ochraceoroseus*, *A. minisclerotigenes*, and *A. nomius.*	Group 1	Carcinogenic, immunosuppressive, teratogenic, genotoxic renal lesions, hepatotoxic, hepatitis, hemorrhage, neuronal degeneration, and cognitive impairment.
Ochratoxin A	*A. carbonarius*,* A. ochraceus*,* A. westerdijkiae*,* A. niger*,* P. verrucosum*, and *P. nordicum*,	Group 2B	Carcinogenic, nephrotoxic, genotoxic, immunosuppressive, causes upper urinary tract disorders, dopaminergic neuronal degeneration, and Parkinson- like neurodegenerative changes.
Fumonisins (FB_1_ and FB_2_)	*F. verticillioides*,* F. proliferatum*, *F*. *sacchari*,* F. fujikuroi*, and *A. niger*	Group 2B	Carcinogenic, nephrotoxic, hepatotoxic, immunosuppressive, myelin injury, cognitive dysfunction, and disrupted sphingolipid metabolism.
Zearalenone	*F. graminearum*,* F. equiset*, *F. culmorum*,* F. semitectum*,* and* *F. incarnatum*	Group 3	Carcinogenic, reproductive effects, hormonal imbalance, teratogenic, memory deficits, cognitive deficits, and hippocampal neuronal injury.
Trichothecenes (DON, T-2, NIV, and HT-2)	*F. langsethiae*,* F. graminearum*, *F. culmorum*,* F. sporotrichioides*, and *F. poae.*	Group 3	Genotoxic, vomiting, immunosuppressive, nausea, diarrhea, reproductive disorder, hepatotoxic, anemia, hypotension, alimentary toxic aleukia, memory impairment, and hippocampal degeneration.

Group 1: established human carcinogen, Group 2B: possible human carcinogen, Group 3: not classified

### Ochratoxins

Ochratoxins (OTs) are a group of widespread secondary fungal metabolites commonly found in food and feed, primarily produced by the *Aspergillus* species, including *A. carbonarius*, *A. ochraceus*,* A. westerdijkiae*, and *A. niger*, as well as by *Penicillium* species, particularly *P. verrucosum and P. nordicum* (Gil-Serna et al. 2015; Wang et al. 2022a, 2022b). OTA has been widely detected in cereals such as barley, wheat, oat, and rice (Li et al. 2021a, 2021b; Hassan et al. 2022; Cantero-Gómez et al. 2026), spices (Chen et al. 2023), coffee and tea (Prakasham et al. 2023), fruit and fruit juice (Aroud et al. 2021), wine (De Jesus et al. 2017), beer (Silva et al. 2020), as well as animal-derived food products (Vlachou et al. 2022; Annunziata et al. 2025). However, there is a lack of adequate information regarding the contamination of food and animal feed by OTB and OTC. These mycotoxins are commonly found alongside OTA in agricultural products (Heussner and Bingle 2015; Guo et al. 2023). Altafini et al. (2021) investigated the incidence of OTA in cheeses obtained from the metropolitan city of Bologna, Italy. OTA was found in 7 out of 51 analysed samples with concentrations ranging from 1.3 to 22.4 µg/kg. OTA has also been detected in wheat and wheat products from Algeria (Zebiri et al. 2019). In that study, different wheat products, including semolina, flour, durum, and common wheat, were analysed for OTA. The results indicated that 62 (76.54%) of the total 81 samples were contaminated at concentrations varying from 0.84 to 34.75 µg/kg. Also, Kumar et al. (2020) reported the presence of OTA in whole-wheat samples from different Malaysian markets. The authors confirmed that the OTA contents in the food varied from 0.15 to 2.11 µg/kg. Furthermore, Omotayo et al. (2019b) examined the presence of OTA in ginger collected during the summer and winter seasons in the Northwest Province of South Africa, and the results revealed that OTA levels of ginger in both summer and winter ranged from 0.0968 to 3.309 and 0.0960–3.395 µg/kg, respectively. Similarly, Wei et al. (2017) reported the level of OTA ranging from 0.2 to 8.8 µg/kg in dried fruits purchased from different markets in China. These levels were found to exceed the 2 µg/kg permissible limit set by the EU (EC, 2022) for OTA in dried fruits.

OTA is highly toxic to both humans and animals, with its primary target being the kidneys. It disrupts protein synthesis by inhibiting phenylalanine-tRNA synthetase, leading to the accumulation of damaged proteins. OTA also induces oxidative stress, inflammation, and apoptosis in renal cells (Liu et al. 2022). Additionally, it suppresses the immune system, affects lymphocytes, and causes hepatotoxicity through lipid peroxidation and mitochondrial dysfunction (Niaz et al. 2020; Liu et al. 2022). Hence, consuming OTA-contaminated food and feed can lead to nephrotoxicity, immunotoxicity, neurotoxicity, hepatotoxicity, and teratogenic effects (Liu et al. 2022; Vlachou et al. 2022). OTA has been linked to Balkan endemic nephropathy (BEN), a kidney disease characterized by interstitial tubule nephritis, and an increased prevalence of cancers in the ureter, kidneys, urinary bladder, and pelvis across Eastern Europe (Stoev 2017; Malir et al. 2021). Increased exposure to OTA has also been associated with nephropathies, such as chronic kidney disease in Morocco, affecting around 2 million people, particularly young individuals (Filali et al. 2002). Ganesan et al. (2021) documented DNA damage and increased ghost cells in Caco-2 cells exposed to OTA. OTA also inhibits Nrf2, inducing oxidative stress that damages DNA and causes mutations (Wu et al. 2019).

Among farm animals, swine are the most vulnerable to OTA accumulation with subsequent kidney damage (Ráduly et al. 2023). As confirmed by Tkaczyk et al. (2021), pigs experienced a gradual development of mycotoxin-induced porcine nephropathy after consuming feed with an OTA concentration of 1,000 µg/kg. It was further reported that even small amounts of OTA could cause symptoms such as polyuria or polydipsia in pigs, while higher levels could induce severe reactions like vomiting, diarrhea, and even fatality (Tkaczyk et al. 2021). The effects of OTA on poultry are frequently characterized by a range of recognizable symptoms, such as reduced egg production, abnormal weight loss, low-quality eggshells, stunted growth, as well as damage to the kidney and liver (Tahir et al. 2022; Sun et al. 2023). Chickens exposed to feed contaminated with 250 µg/kg OTA showed a decrease in feed intake, while those exposed to 1,000–5,000 µg/kg showed symptoms of ochratoxicosis, such as decreased levels of protein, globulin, albumin, potassium, and triglyceride proportions (Wang et al. 2009). The administration of OTA (5,000–20,000 µg/kg) to pregnant Swiss albino mice caused teratogenic and embryotoxic effects (Stoev 2022). The most severe effects were observed at 20,000 µg/kg between days 7 and 12 of pregnancy, with malformations predominantly found in the craniofacial structures of newborn mice. However, lower levels of OTA (5,000 and 10,000 µg/kg) resulted in weaker or no effects at all. Hence, the IARC (1993) has categorized OTA as a possible human carcinogen (Group 2B).

### Fumonisins

Fumonisins (FBs) are mycotoxins that are produced naturally in agricultural products by various pathogenic fungi in the genera *Fusarium* and *Aspergillus*, particularly *F*. *verticillioides*,* F. proliferatum*, *F*. *sacchari*, *F. fujikuroi*, and *A. niger* (Rheeder et al. 2002). A total of 28 homologues of FB, namely fumonisins A, B, C, and P, have been identified (Mihalcea and Amariei 2022). Generally, subgroup B (FB), which includes FB_1_, FB_2_, and FB_3_, is the most prevalent, among which FB_1_ is the most toxic. *Fusarium* spp, the prominent FB producers, are widespread and can invade various food and feedstuffs (Gao et al. 2023). The global prevalence of FBs in food and feed has been thoroughly reviewed (Gruber-Dorninger et al. 2019; Gao et al. 2023), with the most susceptible FB-infected foods being those made from maize and maize-based products (Mngqawa et al. 2016; Mihalcea and Amariei 2022). Apart from their existence in maize and maize products, they have been recorded in several other grains such as sorghum, wheat, barley, rice, oat, and millet (Jedidi et al. 2021; Phan et al. 2023), and other agricultural products, including spices and medicinal plants (El Darra et al. 2019), dried fruits (Chalyy et al. 2021), as well as meat and meat products (Zhao et al. 2015). Mngqawa et al. (2016) investigated the contamination of FB_1_ in home-grown maize produced by rural subsistence farmers in two South African regions (Vhembe and Gert Sibande District Municipalities) over two seasons (2011 and 2012). In both seasons, 92% of samples from Vhembe District Municipality and 47% from Gert Sibande District Municipality contained FB_1_, with levels up to 8,514 and 18,924 µg/kg, respectively. Furthermore, Jedidi et al. (2021) examined the prevalence of FBs and other fungal toxins in different cereals (wheat, maize, and barley) from Tunisia. They reported that FBs (FB_1_ and FB_2_) occurred in 40% of barley, 20.83% of wheat, and 57.14% of maize samples, at levels above the EU maximum limit (1000 µg/kg). This suggests a considerable health risk in humans. This group of mycotoxins has also been reported in animal feed such as fish feed (Rokvić et al. 2020), dairy feed (Changwa et al. 2021), poultry feed (Mokubedi et al. 2019), and pig feed (Castell et al. 2025).

Fumonisins (FBs) exert toxicity mainly by inhibiting ceramide synthase and disrupting sphingolipid metabolism, leading to cellular dysfunction, apoptosis, and damage to cell membranes (Lumsangkul et al. 2021; Tardieu et al. 2021). These disruptions result in hepatotoxicity, gastrointestinal and neurological effects, and may contribute to carcinogenicity following long-term exposure in humans and animals (Wangia-Dixon et al., 2020; Kang’Ethe et al., 2017). FB_1_ is the most extensively studied due to its widespread occurrence and significant toxicological relevance. Consumption of FB_1_-contaminated foods has been linked to high rates of esophageal cancer in regions such as northeast Italy, Transkei region (now Eastern Cape in South Africa, Iran, China, and among black people in Charleston, South Carolina in the United States (Soriano and Dragacci 2004; Zain 2011; Alizadeh et al. 2012; Misihairabgwi, et al., 2017). Esophageal cancer is primarily impacted by FB_1_ because the esophageal tissue lacks protective mechanisms present in other parts of the digestive tract, rendering it more susceptible to direct carcinogenic effects from compounds like FB_1_ (Khan et al. 2018). A study by Yu et al. (2021) showed that even low FB_1_ concentrations promote cell proliferation and migration, increasing cancer marker expression and transforming esophageal epithelial cells into cancer cells. Studies by Marasas et al. (2004) and Missmer et al. (2006) in South Africa and the United States revealed that women exposed to high FB_1_ levels during early pregnancy are at increased risk of having offspring with neural tube defects. Additionally, FB_1_ can contribute to heart failure by affecting myocardial contractility, potentially leading to idiopathic dilated cardiomyopathy (Stoev 2015; Chen et al. 2021a, 2021b).

**Fig. 1:**
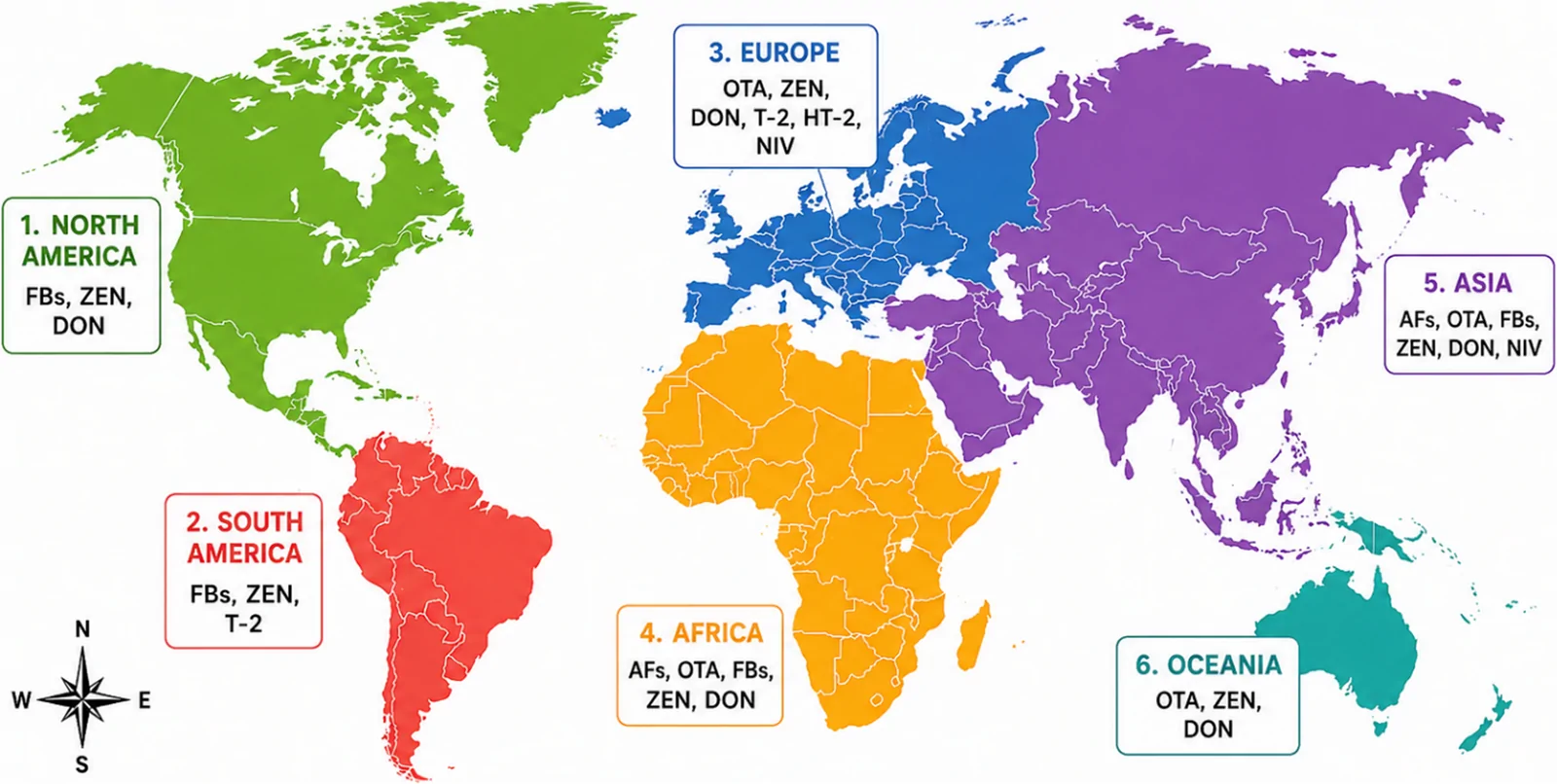
Global distribution of major mycotoxins in food and feed commodities (Binder, 2007; Gruber-Dorninger et al. 2019)

Beyond their toxicity in humans, FBs cause severe adverse effects in animals, including pulmonary oedema in pigs, hepatotoxicity and nephrotoxicity leading to cancer in rats, and equine leucoencephalomalacia in horses (Liu et al. 2019; Odukoya, 2021). Experimental studies have shown that FB_1_ elevates levels of p53-null mouse embryo fibroblasts and increases 8-hydroxy-2′-deoxyguanosine (8-OH-DG) in rat glioma cells, indicating oxidative DNA damage and genomic instability (Mobio et al. 2003; Chen et al. 2021a, 2021b). Immunotoxic effects of FB_1_ have also been widely reported across animal species, with evidence of thymocyte apoptosis, reduced spleen weight, and suppressed splenocyte activity in rodents and avian models following exposure (Abbès et al. 2016; Keck and Bodine 2006; Zhu and Wang 2022). In addition, FB_1_ has been associated with reproductive toxicity, including fetal hypoplasia, reduced maternal feed intake and body weight, and congenital malformations in offspring, as well as impaired sperm production and reduced fertility in male animals (Abbès et al. 2016; Chen et al. 2021a, 2021b; Lumsangkul et al. 2019). Consequently, IARC has classified FB_1_ as a Group 2B carcinogen, underscoring its significance as a food safety concern and a threat to both animal and human health. The global occurrence of FBs and other major mycotoxins (AFs, ZEN, DON, OTA, and T-2/HT-2 toxins) is shown in Fig. 1.

### Zearalenone

ZEN is a primary metabolite produced by *Fusarium* species, including *F. graminearum* (*Gibberella zeae*), *F. equiseti*, *F. culmorum*, *F. semitectum*, and *F. incarnatum* (Rai et al. 2020). The presence of ZEN has been reported in cereals, mainly wheat, maize, barley, and their derived products globally (Stanciu et al. 2019; Rai et al. 2020; Jorquera-Pereira et al. 2021). According to a survey conducted by the European Food Safety Authority (EFSA) panel on mycotoxins in the food chain (EFSA 2011), ZEN was found in 33% of maize samples, with a mean level of 15 µg/kg. Pleadin et al. (2017) examined the presence of ZEN in 415 samples of barley, maize, soybean, and wheat sourced from various farms in four Croatian regions (the eastern, northern, western, and central regions) between 2014 and 2015. The authors discovered that the maximum levels of ZEN in maize, wheat, barley, and soybean in the 2014 season were 3,217, 982, 101, and 89 µg/kg, respectively. In the 2015 season, the maximum concentrations recorded were 19, 874.6, 230, and 121 µg/kg, respectively. ZEN has also been found in animal-derived foods such as eggs, meat, and milk (Iqbal et al. 2014; Falkauskas et al. 2022). Additionally, the prevalence of ZEN and its metabolites has been documented in lamb, pig, and chicken liver samples collected from the Valencian community in Spain (Llorens Castelló et al. 2022).

More importantly, the occurrence of ZEN in animal feed and feedstuffs has been documented globally. Recent research by Wang et al. (2022a, 2022b) investigated the levels of ZEN in 514 poultry feeds obtained from Guangdong, Guangxi, and Hainan provinces in Southern China. The findings confirmed that all the feed samples contained ZEN, with average levels of 2094.1, 14.3, and 8.73 µg/kg in Guangdong, Guangxi, and Hainan, respectively. The study further revealed that more than half (56.9%) of the 167 samples from Hainan exceeded the country’s maximum acceptable limit of 500 µg/kg for ZEN in animal feeds. A similar report of ZEN in 77 South African dairy cattle feeds and feedstuffs by Changwa et al. (2021) also showed high ZEN levels in the cow meals, ranging from 96.7–1,793.7 µg/kg, exceeding the regulatory limits of 500 µg/kg set for South African dairy cattle feeds. In another study, Glamočić et al. (2019) examined 48 silage samples from various dairy farms in Vojvodina, Serbia, for ZEN, along with OTA and AFB_1_. Their results revealed that the ZEN concentrations in the silage samples reached up to 538 µg/kg, slightly exceeding the EU’s maximum limit of 500 µg/kg.

ZEN toxicity was first reported in the late 1920 s in the Midwestern United States following cases of hyperestrogenism in pigs fed moldy grains (McNutt et al. 1928). Since then, ZEN has drawn significant attention due to its adverse effects on human and animal health. Although its toxic mechanism in humans is not fully understood, evidence suggests that ZEN acts as an endocrine disruptor and can also modulate both innate and adaptive immune responses, potentially increasing susceptibility to infections and disease (Kowalska et al. 2016; Woźny et al. 2019). Moreover, ZEN has been reported to disrupt the human reproductive system by affecting the production and secretion of sex hormones like testosterone, estradiol, and progesterone (Liu et al. 2014; Rai et al. 2020). Many studies have also confirmed the susceptibility of children who consumed ZEN-contaminated foods to early puberty (Rai et al. 2020). ZEN can also initiate hyperestrogenic syndrome in humans (Rai et al. 2020), with symptoms including weight gain, decreased sex drive, irregular menstrual periods, fatigue, and breast enlargement.

Various studies have reported that ZEN poses a threat to various animal species, including poultry, swine, horses, and cattle (Liu and Applegate 2020; Rai et al. 2020; Li et al. 2021a, 2021b). When animals are exposed to ZEN, the toxin is swiftly absorbed, distributed to multiple organs, and excreted quickly, resulting in its detection in animal by-products (Knutsen et al. 2017). ZEN enhances LPS-Induced endoplasmic reticulum and oxidative stress in bovine mammary epithelial cells (Fu et al. 2022). After pregnant sows were exposed to 200, 500, and 1000 µg/kg of ZEN, the number of healthy follicles in the F-1 prepubertal gilts was reduced (Schoevers et al. 2012). Conversely, female pigs fed a 1300 µg/kg ZEN diet experienced decreased levels of platelets, globulin, high-density lipoproteins, and hemoglobin in their serum (Jiang et al. 2010). Recently, Yousef et al. (2023) determined the negative impact of ZEN on buffalo embryo production by exposing buffalo sperm and oocytes to different concentrations of ZEN during maturation. The findings revealed a decrease in the number of acrosome-intact sperm following exposure to concentrations below 100 ng/mL. Additionally, the maturation rate of buffalo oocytes decreases drastically, accompanied by a higher rate of degeneration. Based on these outcomes, the authors suggested that buffaloes should be considered sensitive to ZEN exposure. However, ZEN’s carcinogenicity is still unclear, as the IARC has classified it as a Group 3 carcinogen, indicating that the compound is not considered a human carcinogen (IARC, 1993).

### Trichothecenes

Another group of fungal toxins, TCTs, are sesquiterpene compounds produced via the terpenoid biosynthesis pathway by *Fusarium* spp., including *F. langsethiae*,* F. graminearum*, *F. culmorum*, *F. sporotrichioides*, *F. poae*, and many more *Fusarium* spp. (Valle-Algarra et al. 2011; Wegulo 2012; Nazari et al. 2018). More than 200 TCTs have been discovered, and they are classified into four types (A, B, C, and D) based on their chemical structure (Proctor et al. 2020). Type A-TCT, including T-2 toxin and its major hydrolysis product, HT-2 toxin, and type B-TCTs such as DON and NIV are the most toxicologically significant and frequently found TCTs in foods such as barley, maize, rice, oats, wheat, nuts, and even spices and feeds (Mokubedi et al. 2019; Jorquera-Pereira et al. 2021; Changwa et al. 2021), with a high incidence rate in Europe, Asia, and Africa (Streit et al. 2013). Between the period 2017 to 2019, Kosicki et al. (2020) conducted a study to examine the occurrence of four TCTs (DON, ZEN, T-2, and HT-2 toxins) in Polish rye, with results revealing that DON, T-2, and HT-2 toxins had the highest prevalence in the rye samples, at 90, 63, and 57%, respectively. The average levels of these mycotoxins were 28.8, 0.98, and 2.98 µg/kg, whereas the highest recorded levels were 354.1, 6.63, and 29.8 µg/kg, respectively. In another study, Gab-Allah et al. (2021) investigated the prevalence of type B-TCTs (NIV and DON) in 67 cereal samples, including corn, rice, and wheat, from Korea between 2019 and 2020. The presence of these mycotoxins was observed in 86.5 and 94% of the samples, with maximum concentration levels of 234.1 and 1223.3 µg/kg, respectively. A study by Dong et al. (2022) revealed the presence of NIV in staple foods, including maize, rice, and wheat, from Sichuan Province, China.

Also, the incidence of TCTs in animal feed and feedstuff have been reported worldwide. According to Pack et al. (2021), DON and ZEN were the predominant *Fusarium* toxins contaminating swine feeds sourced from the United States, with incidence of 94 and 57%, respectively. Zaworska-Zakrzewska et al. (2020) reported the occurrence of T-2 and HT-2 toxins in 18 soybean seed samples intended for weaned pig feed. The study showed that 10 and 9 samples were contaminated with T-2 and HT-2 toxins, respectively, with T-2 concentrations ranging from < 0.6 to 181 µg/kg and HT-2 levels from < 2.0 to 288 µg/kg. Likewise, Reisinger et al. (2019) reported elevated NIV levels in maize silage samples collected across Europe from 2014 to 2018. Their findings showed that 59.5% of the samples contained NIV, with an average concentration of 113 µg/kg.

Trichothecenes (TCTs) cause multiple adverse effects in humans and animals, including neuroendocrine disturbances, growth retardation, anorexia, and emesis (Mahato et al. 2022). After absorption, they rapidly affect intestinal epithelial cells and other rapidly proliferating cells, thereby impairing tissue growth and intestinal barrier function. Significant mycotoxins, including TCTs, doses, exposure duration, route of exposure, and their impacts on animal health, are highlighted in Table 2. DON, the most prevalent TCT, exerts toxicity mainly by inhibiting protein synthesis through ribosomal binding, leading to oxidative stress, immunomodulation, and gastrointestinal and neurological disorders (Pestka 2010). Frequently observed responses to DON poisoning in animals include vomiting, nausea, and weight loss (Jia et al. 2023). Jia et al. (2023) demonstrated that DON exposure induces gut flora disruption, dysfunction, and organ damage in broilers and pigs. This mycotoxin has also been linked to decreased milk production in dairy animals and feed rejection in swine and cattle (Munkvold et al. 2019).

**Table 2:** Major mycotoxins, doses, duration of exposure, exposure route, and their effects on animal health

Mycotoxins	Animals	Doses Exposure route	Duration of exposure	Health effects	References
AFB_1_	Cattle Poultry Mice	33.5 mg/kg Ingestion 0.10–0,15 mg/kg Ingestion 0.3 mg/kg bw Ingestion 0.37–1.5 mg/kg bw Ingestion	3–12 h 7–21 days 28 days 30 days	Anorexia, depression, photosensitization, diarrhea, and death. Increased kidney and liver weight. Caused severe kidney deterioration accompanied by tubular necrosis and congestion. Aggravated non-regenerative anemia, reduced leucocyte functionality, and suppressed antibody production. Induced persistent anemia, decreased body weight, and lowered the levels of red blood cells and hemoglobin. Reactive oxygen species and apoptotic cells in the red blood cells showed a substantial elevation. Initiated oxidative stress-induced liver damage. Caused structural impairment of mitochondria, reduced mitochondrial membrane potential, and enhanced expression of cytoplasmic cytochrome c protein.	Umar et al. (2015) Prakoso (2019) Rajaura et al. (2023) Xu et al. (2021)
OTA	Piglets Rabbit Mice	0.4–0.8 mg/kg Ingestion 0.2 mg/kg Ingestion 20 mg/kg Ingestion	42 days 7 days 7–12 days	Induced deterioration of hepatic and proximal tubule epithelial cells, triggered oxidative stress, and resulted in histological alterations in the kidneys and livers. Suppressed the levels of Nrf2 and HO-1, which leads to peroxide reaction. Teratogenic and embryotoxic effects were reported, as well as deformities in the craniofacial features of newborn mice.	Zhang et al. (2016) Zhang et al. (2022) Stoev (2022)
FBs (FB_1_ & FB_2_)	Piglet Duck Pig Chicken	3.7 mg/kg Ingestion 0.04 mg/kg Inhalation 11.8 mg/kg Ingestion 20 mg/kg Ingestion	28 days 3 days 63 days 9 days	Alterations in the intestine and heart appeared. Caused growth retardation, developmental abnormalities, and neural tube defects in the duck embryo. Resulted in a digestive microbiota imbalance. Modifications of sphingoid bases. Ceramides and sphingomyelins were detected in the liver.	Tericiolo et al. (2019) Lumsangkul et al. (2021) Burel et al. (2013) Tardieu et al. (2021)
ZEN	Laying hens Mice Pig Piglet Female mice	0.4 mg/kg Ingestion 5–200 mg/kg Ingestion 40 mg/kg bw Ingestion 0.2 & 0.4 mg/kg Ingestion 3 mg/kg Ingestion 30 mg/kg bw Injection	18 days 28 days 28 days 7 days 28 days 0–12 days	Increased relative weight of the oviduct and ovary caused deterioration and shrinkage of the ovaries. Interfered with the secretion of sex hormones β-endorphin and progesterone. Caused renal edema and increased the content of creatinine and urea. Reduced testis, seminal vesicles, and prostate weights. Diminished sperm count and motility. Triggered ovarian follicle atresia and apoptotic-like changes in the granule cells. Intensified cell growth in the uterus and oviduct. Caused vulvar malformation and disorder of the level of serum hormones. Suppressed immune responses. Induced apoptosis, dysfunction, and oxidative stress in the kidney.	Chen et al. (2017) Ling et al. (2019) Salah-Abbés et al. (2009) Obremski et al. (2003) Su et al. (2018) Liang et al. (2015)
TCTs DON HT-2/T-2 T-2	Mice Broiler chicken Dog Mice Mice Ruminants Pig Turkey Duck Laying Hens	12.5 mg/kg Ingestion 2.5 mg/kg Ingestion 1–5 mg/kg Ingestion 4.5–10 mg/kg Ingestion 3 mg/kg Ingestion 1 mg/kg Ingestion 1 mg/kg Ingestion 0.3 mg/kg Ingestion 0.029 mg/kg 0.04–0.048 mg/kg 0.04 mg/kg 0.12 mg/kg	0–3 h 6 h 35 days 14 days 14 days 2–6 h 1 day 1 day 1 day 1 day 1 day 1 day	Reduced feed intake and proinflammatory cytokines. Loss of appetite. Resulted in small intestine alteration and weight loss of various organs, including the liver, spleen, and heart. Caused feed intake reduction and vomiting. Caused a 35% decrease in feed intake, accompanied by a 5% decline in body weight. Induced anorectic response and secretion of plasma 5-HT and SP. Caused a decrease in feed consumption. Resulted in alteration of serum protein, hematological, and gastrointestinal parameters. Caused immunological and hematological effects. Triggered mucosal oral damage. Loss of appetite. Induced infertility and reduced egg production.	Tominaga et al. (2016) Wu et al. (2014) Awad et al. (2011) Hughes et al. (1999) Leung et al. (2007) Zhang et al. (2018) Sheng et al. (2018) EFSA (2017)

Generally, toxicity data for T-2 and HT-2 in humans are limited compared to the other mycotoxins discussed in this review. Nevertheless, studies have shown that these mycotoxins may impair protein synthesis and are associated with neurotoxic, hepatotoxic, haematotoxic, immunotoxic, and reproductive effects (Dai et al. 2019; Janik et al. 2021). More so, T-2 and HT-2 are known to cause emesis, growth retardation, severe inflammatory reactions, delay bone marrow and spleen regeneration, as well as alimentary toxic aleukia (ATA) in animals (Liu et al. 2016; Ekwomadu et al. 2021; Janik et al. 2021). Dietary exposure to T-2 and HT-2 has been shown to reduce immunological responses in swine and cause oral lesions in poultry (EFSA, 2017). A case study involving exposure of sheep to T-2 toxin reported adverse effects, including the death of 19% (190) of the total 1000 ewes, at an average concentration of 35 µg/kg (Ferreras et al. 2013). In a recent study by Nayakwadi et al. (2020), 18 young goats (2–3 months old) were fed T-2 toxin (10,000 and 20,000 µg/kg) contaminated feed. Both groups showed reduced feed intake, weight loss, and lethargy. The treated goats also demonstrated lowered hemoglobin, total leukocyte, and thrombocyte counts, in addition to hematological changes.

Dietary exposure to NIV-contaminated cereals has been studied in ecological investigations of regions with high rates of gastric and esophageal cancers. Hsia et al. (2004) documented relatively high NIV levels (584–1,780 µg/kg) in barley, wheat flour, and maize samples sourced from Linxian and Henan provinces in China, areas known for elevated rates of esophageal and gastric cardia cancer. However, a direct causal relationship between NIV exposure and these cancers in humans has not been established. Kashin-Beck disease (KBD) is an endemic degenerative osteoarthropathy affecting children and adolescents in several regions of China. The etiology of KBD is considered to be multifactorial in nature (Bianco et al. 2012) and has been hypothesized to include dietary mycotoxin exposure, including NIV. Also, experimental animal studies have linked exposure to NIV with oxidative stress, DNA damage, and reduced oocyte quality in mice (Wang et al. 2021). Hence, the IARC has classified NIV and other TCTs as Group 3 agents (not classifiable as to their carcinogenicity to humans) due to insufficient evidence of its carcinogenic effects in humans (IARC, 1993).

## Co-occurrence of mycotoxins in food and feed

Despite the extensive focus on individual mycotoxins in commodities over the past decades, there is a growing awareness of the need to understand the co-occurrence of multiple mycotoxins in food and feed. It is important to mention that most farm products can be infected by an array of toxigenic or individual fungi with multiple toxin-producing potentials. For instance, *A. flavus* can produce B-type AFs (AFB_1_ and AFB_2_) and cyclopiazonic acid, while A. *Parasiticus* produces both B-type (AFB_1_ and AFB_2_) and G-type (AFG_1_ and AFG_2_) AFs. *A. niger* is known for the biosynthesis of FBs (FB_2_ and FB_3_) and OTA. Furthermore, *F. graminearum* can produce mycotoxins such as DON and ZEN in agricultural commodities.

As a result, there is a potential for the accumulation of multiple mycotoxins within the same matrix, which can have greater or lesser health consequences than exposure to individual toxins (Smith et al. 2016; Castells et al. 2019).

### Co-occurrence of mycotoxins in food

Numerous worldwide studies have documented the assessment of a considerable variety of mycotoxin co-occurrence in several food or feed commodities (El Darra et al. 2019; Muñoz-Solano and González-Peñas 2023; Poapolathep et al. 2026), all posing an infinite number of distinct mycotoxin combinatory effects, for which no efficient assessment strategy has yet been established. In their study on the worldwide occurrence of mycotoxins in cereal grains and their products, Smith et al. (2016) identified a total of 127 possible mycotoxin combinations, with DON + ZEN, AFs + FBs, AFs + OTA, and FBs + ZEN being the most notable combinations. Regarding African samples, the same systematic review found 26 common combinations, with AFs + OTA comprising 35% of the 14 publications analysed and the AFs + OTA+ZEN combination making up 29% of the total. In a recent study done by Daou et al. (2023), the potential public health risk associated with the concurrent presence of AFB_1_ and OTA in about 198 samples of herbs, spices, and nuts sourced from Lebanon were investigated. AFB_1_ was detected in 20.4, 100, and 98.6% of the herbs, spices, and nuts, respectively, with mean concentrations of 0.27, 0.97, and 0.40 µg/kg, respectively. Similarly, OTA was found in 44.4 and 100% of the herbs and spices, with mean concentrations of 1.81 and 38.8 µg/kg, respectively.

The co-occurrence of mycotoxins in raw wheat grains has been documented in China (Zhao et al. 2021a, 2021b). From a total of 338 wheat samples, there were 44 mycotoxin combinations, with DON and its derivatives (DON + 3-ADON + 15-ADON) being the most common combination, occurring in 11.2% (38/338) of samples, followed by DON + 3-ADON + NIV (7.7%), DONs + NIV + ZEN + FBs (7.4%), DONs + NIV + ZEN (6.2%), DONs + NIV (5.0%), DON + 3-ADON (4.7%), and DON + 3-ADON + NIV + ZEN (3.6%). In Southern Brazil, Santos et al. (2021) discovered a significant prevalence of mycotoxins in 200 wheat flour samples collected between 2013 and 2014. In 2013, 100% of the positive samples contained DON + ZEN mycotoxins; however, in 2014, the most frequent mycotoxin combination in the positive samples was DON + T-2 toxin (27%). The three-toxin combination (ZEN + T-2 toxin + DON) was detected in 2% of the wheat samples. Kara et al. (2015) investigated the co-occurrence of AFs and OTA in 24 commercial maize flour samples from Turkey. They discovered that 37.5% of maize flour samples were tainted with both toxins.

### Co-occurrence of mycotoxins in feeds

In earlier years, Streit et al. (2013) conducted a comprehensive investigation of multi-mycotoxin (AFs, DON, FBs, OTA, and ZEN) in 17,316 feed and feed raw materials, focusing on Asia and Europe. The findings from the 8-year survey indicated that 72% of the samples contained at least one mycotoxin, whereas co-contamination with multiple mycotoxins was observed in 38% of the samples. Likewise, Muñoz-Solano and González-Peñas (2023) documented the simultaneous presence of several mycotoxins in 400 feeds for pigs, sheep, cattle, and poultry in Navarra, Northern Spain. They discovered that 63.5% of the feed samples contained two to five detectable mycotoxins, with DON, ZEN, AFB_1_, and AFB_2_ being the most prevalent. Among the different types of animal feed, poultry feed had the highest occurrence of multiple mycotoxins (70%), followed by sheep feed (63%), cattle feed (62%), and pig feed (59%). A global study assessing mycotoxin co-occurrence patterns in feed samples found that for maize samples and finished feed, combinations of DON, FBs, and ZEN were most frequently observed. In addition, DON + ZEN, DON + FBs, and ZEN + FBs with AFB_1_+FBs were also observed in significant proportions in the maize samples (Gruber-Dorninger et al. 2019). Multi-mycotoxin occurrences have been detected in 45 maize-based feeds for layers, broilers, and dairy cattle sourced from smallholder farmers in São Paulo and Santa Catarina, Brazil (Fanco et al., 2019). According to the authors, FB + ZEN + DON was the most frequent co-occurring mycotoxin, found in 15.6% of the samples, followed by FB + DON (13.3%). Other mycotoxin combinations were AF + FB (6.7%), FB + ZEN (4.4%), AF + FB + DON (2.2%), AF + FB + ZEN + DON (2.2%), and AF + FB + OTA + ZEN + DON (2.2%).

Awapak et al. (2021) reported the prevalence of different mycotoxin combinations in feed destined for dairy cattle in Thailand, with ZEN + FB₁ observed in approximately 66% of the samples, followed by ZEN + DON, which was observed in approximately 57% of the samples. Other combinations included ZEN + AFB₁ (approx. 34%), FB₁ + AFB₁ (31%), and FB₁ + DON (approx. 29%). An analysis of mycotoxin contamination in 91 South African poultry feed samples conducted by Mokubedi et al. (2019) revealed the co-existence of several mycotoxins in the feed samples, with 67% having three mycotoxins (DON + FB₁ + ZEN) and 26% having four toxins (AF + FB_1_ + DON + ZEN) in combination. From 2016 to 2017, 1569 feed ingredients and complete feeds from China were found to be contaminated with various combinations of ZEN, DON, and AFB_1_. The study reported that AFB_1_ + DON, ZEN + DON, AFB_1_ + ZEN, and AFB_1_ + ZEN + DON co-occurred in feed ingredients in the range of 75.0–100, 76.9–100, and 76.9–100%, respectively. Meanwhile, the co-occurrence of AFB_1_ + ZEN, AFB_1_ + DON, ZEN + DON, and AFB_1_+ ZEN + DON in the complete feed varied from 96.4 to 100, 96.4–100, 97.9–100, and 96.4–100%, respectively. Preventing animal and human exposure to individual and multiple mycotoxins is crucial because the presence of one or a combination of these harmful substances in food and feed can have harmful effects on human and animal health.

## Combined effect of mycotoxins on human and animal health

Multiple mycotoxins co-occurring in food and feed is a complex issue that necessitates a better understanding of their combined effects on animal and human well-being. Moreover, depending on the mycotoxin types and concentrations, interactions among them can be synergistic, additive, or antagonistic, contributing to overall toxicity. These effects are somewhat combined yet still related to individual toxins’ metabolism, toxicodynamics, and toxicokinesis (Smith et al. 2016; Ráduly et al., 2023; Kifer et al. 2020). Synergism is reflected by the toxicity of the mixture being greater than the sum of its individual components (Alassane-Kpembi et al. 2013; Smith et al. 2016), whereas ‘potentiation’ is a type of synergy where mycotoxins do not exert individual effects but display a significant collective effect when combined (Kpembi and Oswald 2016; Smith et al. 2016). Additive effects occur when mycotoxins act together without interaction, and their total effect equals the sum of individual effects (Smith et al. 2016). In contrast, antagonistic effects show a lower-than-expected response, as observed with ZEN and certain TCTs (Fink-Gremmels, 1999; Kpembi and Oswald 2016). Extensive research employing human and animal cell line models has investigated the interactive effects of mycotoxins on health. These studies have revealed adverse outcomes such as oxidative stress, immune suppression, reproductive impairment, and cytotoxicity, among others (Table 3).

**Table 3:** Interactive effects of mycotoxins on human and animal health

**Mycotoxin combination**	**Effects on human health**	**Model**	**Reference**
AFM_1_+DON/AFM_1_ +ZEN	• Enhanced cytotoxicity	Human intestinal Caco-2 cells	Gao et al. (2016)
DON + NIV + [3-ADON + 15-ADON+ ANIV]	• Cytotoxicity & pro-inflammatory responses	Human intestinal epithelial cells.	Alassane-Kpembi et al. (2015)
DON + NIV	• Increased cytotoxicity and DNA damage	Human intestinal epithelial cells.	Alassane-Kpembi et al. (2013)
DON+FB_1_/DON + T-2/ DON + ZEN/T-2 + DON	• Cytotoxicity	Hematopoietic progenitor’s cells	Ficheux et al. (2012a, 2012b)
FB_1_+ZEN	• DNA fragmentation • Lipid peroxidation • Cell proliferation	Human lymphoblastoid cells Jurkat T	Luongo et al 2006
DON+AFB_1_/DON + ZEN+AFB_1_	• Intestinal cytotoxicity • Mitochondrial dysfunction • Oxidative stress • Impaired pyruvate/energy metabolism	Human Caco-2 intestinal epithelial cell line	Ji et al. 2019
FB_1_+ZEN	• Cytotoxicity	Human epithelial colorectal Adenocarcinoma cells Adenocarcinoma cells Caco-2	Kouadio et al. 2007
DON + ZEN	• Cytotoxicity	Human epithelial colorectal adenocarcinoma cells.	Kouadio et al. 2007
DON + T-2	• Cytotoxicity	Monkey kidney epithelial cells	Ruiz et al. 2011a
AFB_1_+OTA	• Enhanced neurotoxicity	Human SH-SY5Y cells	de Sá et al. 2024
**Mycotoxin combination**	**Effects on animal health**	**Model**	**Reference**
DON + ZEN	• Oxidative stress • Hepatotoxicity • Apoptosis • Inflammation	Zebrafish embryos	Rong et al. (2023)
AFB_1_+OTA + ZEN	• Decreased milk production • Increased liver enzyme activity • Immunosuppression • Decreased antioxidant enzyme activity	Lactating dairy goats	Huang et al. (2018)
DON + ZEN	• Moderation of blood biochemical indices [alanine transaminase, alkaline phosphatase, and albumin (ALB)]	Mice liver tissue & serum	Ji et al. (2017)
AFB_1_+DON/AFB_1_+ZEN/ DON + ZEN/AFB_1_+ DON + ZEN	• Liver damage • Increased mortality	Zebrafish.	Zhou et al. 2017
AFB_1_+DON/AFB_1_+ZEN/ DON + ZEN/AFB_1_+DON + ZEN	• Caused cytotoxicity	BF-2 cells	Zhou et al. 2017
DON + T-2	• Cytotoxicity	Chinese hamster ovary cells CHO-K1	Ruiz et al. 2011b
AFB_1_+OTA	• Kidney injury • Liver injury • Immune inflammation	Pullet (young chicken)	Qing et al. 2022
DON + NIV/DON+FB_1_/ZEN+FB_1_/DON + NIV+ZEN/DON + ZEN+FB_1_/DON + NIV+ZEN+FB_1_	• Intestinal cytotoxicity	Swine jejunal porcine epithelial cells	Wan et al. 2013
AFB_1_+OTA + DON	• Impair liver and oviduct health • Reduced production performance	Broiler breeder hens	Ruan et al. 2023

### Combined effect of mycotoxins on human health

Over the years, studies have documented various synergistic effects among different mycotoxins. For example, combined exposure to DON and ZEN has been shown to exert synergistic toxicity, causing greater reductions in hematopoietic progenitor cell viability and inducing dose-dependent oxidative stress in porcine splenic lymphocytes compared with individual mycotoxin exposure (Ficheux et al. 2012a, 2012b; Ren et al. 2017). Using porcine Leydig cells (testosterone-producing cells in the male testes), Ling et al. (2020) demonstrated that co-exposure to T-2 toxin and its derivatives, including HT-2 and Neosolaniol (NEO), led to reproductive cytotoxicity in a dose-dependent manner. Synergism was observed for T-2 + HT-2, T-2 + NEO, and HT-2 + NEO, but antagonism was observed at higher doses. The authors emphasized that co-exposure to T-2 toxin and its derivatives could negatively impact Leydig cell survival and male reproduction. According to Pinhão et al. (2020), simultaneous exposure to OTA and FB_1_ led to synergistic cytotoxic actions in human kidney (HK-2) and liver (HepG2) cell lines, especially at lower concentrations. The OTA+FB_1_ combination reduced cell viability, implying increased nephrotoxicity and hepatotoxicity potential without enhancing DNA or oxidative DNA damage. Co-exposure to type A TCT toxins (T-2 and HT-2) and diacetoxyscirpenol (DAS) has been associated with immunotoxic synergy in human Jurkat T cells (Wattanasuntorn et al. 2025). As revealed by the authors, the effect of the combined toxins (T-2 + HT-2 + DAS) inhibited T cell viability and enhanced the production of reactive oxygen species (ROS) and apoptosis at a level comparable to that which occurs due to food/feed contamination.

### Combined effect of mycotoxins on animal health

Xue et al. (2010), in their experiment, examined the combined effect of T-2 toxin and OTA on the immunopathology of poultry. Their findings indicated that administering both toxins resulted in a 10.4% reduction in the antibody titres for Newcastle disease. The authors also observed a decrease in the messenger ribonucleic acid (mRNA) expression of interleukin-2 and interferon and physical deformities in the bursa of the liver, spleen, fabricius, and thymus. Similarly, Liang et al. (2015) confirmed that the combination of DON and ZEN induced nephrotoxicity in Female Kunming mice. Current studies assessing more appropriate and accurate cell lines or mathematical modelling for interactive effects of mycotoxins in humans and animals are, however, on the increase (Kifer et al. 2020; Karsauliya et al. 2022), with hopes to bridge gaps in information towards the more precise elucidation of mechanisms of action and establish interactive sub-acute contamination/risk ranges. Sun et al. (2015) showed that exposure to AFB_1_ + ZEA and AFB_1_ + DON induced a synergistic effect on hepatotoxicity in BRL 3 A rat liver cells. In particular, the combined toxins resulted in a decrease in cell viability due to elevated levels of ROS in cells and induction of apoptosis caused by the upregulation of Hsp70, p53, Bax, Caspase-3, Caspase-8 proteins, and downregulation of the anti-apoptotic gene Bcl-2.

Co-exposure to AFs and ZEN in birds has been found to exhibit synergistic effects in reducing laying performance and egg quality (Jia et al. 2016). Similarly, exposure to combined AFB_1_ and ZEN adversely affects fish growth performance, lowers the activity of intestinal digestive enzymes, causes oxidative stress and innate immune inflammation, and leads to histological changes in the intestine, such as shortening of villi and decreased goblet cell density, indicating increased susceptibility to mycotoxin-induced intestinal and immunological injury (Ghafarifarsani et al. 2021). Based on the available evidence, the effects of mycotoxins on human and animal health are dose-, tissue-, and species-dependent, with synergistic interactions being the most commonly observed effect of mycotoxin exposure at low and moderate levels. In human cellular models, increased hepatotoxicity, nephrotoxicity, neurotoxicity, immunotoxicity, and cytotoxicity of intestinal cells were observed in combined exposures, whereas animal experiments confirmed these toxicities at the organism level as developmental toxicity, liver and kidney damage, immune system dysfunction, damage to the intestinal tract, and reduced productivity. This suggests that the risks associated with exposure to natural mycotoxin mixtures may be underestimated by current risk assessment methodologies, which rely solely on single mycotoxins.

## Regulatory standards and maximum permitted levels for mycotoxins in food and feed

To mitigate human and animal health risks from mycotoxin exposure, regulatory authorities worldwide have established maximum permitted levels (MLP) and guidance values that vary by region, commodity, and target consumer groups (Supplementary Table 1). More stringent limits are typically applied to foods intended for infants and other vulnerable populations due to their higher susceptibility to toxic effects. At the international level, the Codex Alimentarius Commission, under the joint auspices of FAO and WHO, provides harmonized reference limits based on toxicological evaluations conducted by the Joint FAO/WHO Expert Committee on Food Additives (JECFA) (Duarte et al. 2010; Meneely et al. 2023). The European Union (EU) has some of the most comprehensive and stringent mycotoxin regulations, through Commission Regulation (EU) 2023/915 (European Commission 2023) and its amendments, which specify maximum levels (MLs) for a wide range of mycotoxins and distinguish between limits for raw materials and processed foods, as well as products intended for infants and young children.

Similarly, in the United States, the Food and Drug Administration (FDA) establishes action levels that guide regulatory enforcement and determine when contaminated food or feed may be subject to rejection or recall. Other countries, including China, Singapore, Malaysia, Japan, India, and a few African nations (Supplementary Table 1), have developed national regulatory frameworks that are largely informed by Codex or EU standards but are tailored to local dietary patterns, staple commodities, climatic conditions, and agricultural practices. Despite extensive efforts, global harmonization of mycotoxin standards remains difficult due to differences in risk assessment, dietary patterns, analytical capacity, enforcement, and socio-economic contexts. Most regulations focus on single mycotoxins, despite frequent co-exposure with additive or synergistic effects, highlighting the need for regular regulatory updates, stronger surveillance, and the integration of climate- and mixture-related evidence to better protect human and animal health.

### Impact of climate change on mycotoxin contamination in food and feed, human and animal exposure, and health risks

**Fig. 2:**
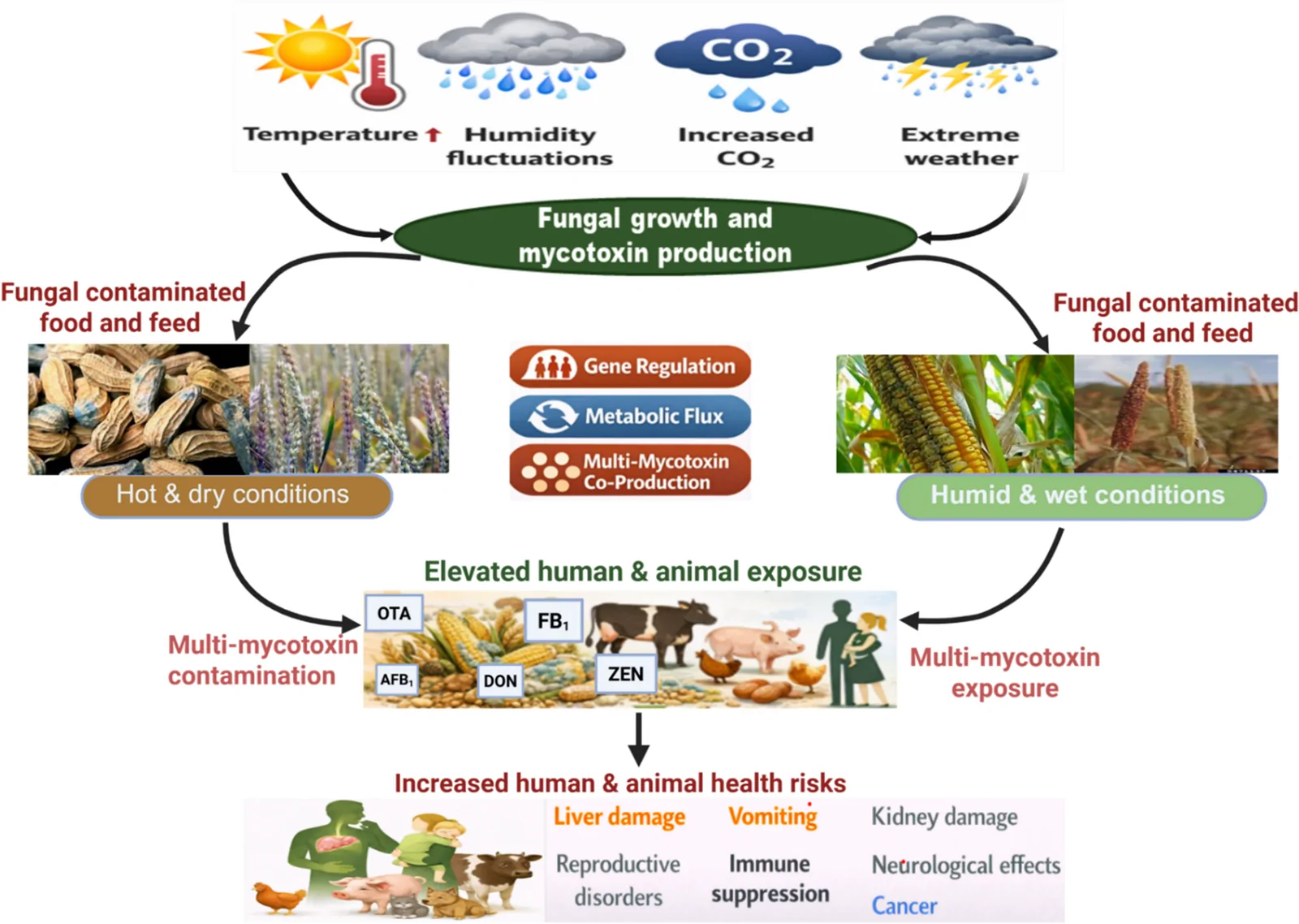
Climate-driven pathways of fungal proliferation, multi-mycotoxin contamination, and escalating human and animal health risks across food and feed systems

Over the past decade, evidence has increasingly shown that climate change is altering the prevalence, distribution, and exposure risk of mycotoxins in food and feed systems (Fig. 2)**.** Elevated global temperatures have altered precipitation patterns, and the increasing frequency of extreme weather events creates environmental conditions that favor the growth and toxin biosynthesis of major mycotoxigenic fungi, including *Aspergillus*,* Fusarium*, and *Penicillium* (Medina et al. 2017; Platzer et al. 2025; Yan et al. 2026). Al-Zaban (2023) further demonstrated that climatic stressors not only intensify fungal colonization but also alter the regulation of mycotoxin biosynthetic genes and associated metabolic pathways, thereby promoting the co-production of multiple mycotoxins. Climate change also affects mycotoxin exposure pathways by altering crop phenology, facilitating the geographic expansion of mycotoxigenic fungi into regions that were previously temperate, and modifying post-harvest storage and handling conditions. Kos et al. (2023) and Casu et al. (2024) reported that climate-driven shifts in flowering and maturation increase crop susceptibility to fungal infection at critical growth stages, while the poleward expansion of *Aspergillus* and *Fusarium* introduces mycotoxin risks into previously unaffected production regions (Kos et al. 2023). In parallel, increased reliance on irrigation as an adaptation to drought, extended storage durations necessitated by market and supply-chain disruptions, and higher ambient humidity during storage and transport further exacerbate contamination risks, particularly in low- and middle-income countries where infrastructure and climate control are often limited (Nji et al. 2022; Herrera et al. 2023).

Climate projections indicate that some tropical regions may warm up to 5.8 °C this century, with more frequent extreme events such as droughts and heavy rainfall. Under + 2 to + 5 °C warming scenarios, models predict a marked increase in mycotoxin risk in agricultural products across different agroecological regions, highlighting increasingly complex future mycotoxin exposure patterns (Perrone et al. 2020). According to the 2019 *Facts and Figures* for the East African Community, the region experienced peak annual rainfall of up to 953 mm in 2018, alongside average maximum and minimum temperatures of approximately 29 °C and 20 °C, respectively (Ankwasa et al. 2021). These warm and humid climatic conditions are highly conducive to fungal proliferation and have been associated with extensive colonization of maize by *Aspergillus* species and elevated AF contamination (Kimanya 2015). More so, Mutiga et al. (2015) evaluated mycotoxin contamination in maize flour from three agroecological zones in western Kenya and reported highly variable AF levels, ranging from 2 to 710 µg/kg, with the most severe contamination observed in drought-affected areas. Observational studies and monitoring data from Europe’s human biomonitoring projects indicate that populations are already exposed to harmful levels of DON, FB_1_, and other mycotoxins at rates exceeding health‑based guidance values, with climate‑related fungal expansion cited as a contributing driver of increased exposure (Alvito et al. 2022). With up to 60–80% of global crop samples thought to contain detectable mycotoxin levels, climate influences are implicated in elevated contamination beyond what would be expected from analytical improvements alone, particularly in staple grains and cereals (Perrone et al. 2020; Nji et al. 2022).

As a result, exposure to mycotoxins through staple foods such as cereals, nuts, and legumes, as well as animal-derived products including milk, meat, and eggs, is likely to increase due to climate-enhanced contamination and carry-over along the food chain. Elevated dietary exposure may intensify the risk of adverse health effects in humans, including immunotoxicity, hepatotoxicity, carcinogenicity, endocrine disruption, and impaired growth and development, particularly among vulnerable populations such as infants and immunocompromised patients (Zain 2011). In animals, increased mycotoxin intake caused by climate change can lead to reduced feed efficiency, growth impairment, reproductive disorders, immune suppression, and decreased productivity, ultimately resulting in significant economic losses (Rehman et al. 2022; Akinmoladun et al. 2025). Overall, climate-driven changes in mycotoxin contamination patterns pose an emerging and complex challenge to food safety and animal health, highlighting the urgent need for climate-aware monitoring systems, innovative mitigation strategies, and adaptive risk assessment frameworks to mitigate future impacts.

## Mycotoxin risk assessment in the era of climate change

Risk assessment is a systematic process used to evaluate potential hazards and quantify associated risks from chemical, microbiological, and nutritional sources in food or feed (Renn et al. 2022). Mycotoxin risk assessment specifically aims to understand the likelihood and levels of mycotoxin contamination and their potential impact on human or animal health. Humans and animals are often exposed to combinations of mycotoxins rather than single compounds due to their natural co-occurrence in food and feed (Fan et al. 2019; Kyei et al. 2022; Liao et al. 2023), while climate change-driven increases in temperature and humidity further promote the growth of toxigenic fungi (Medina et al. 2017). Assessing mycotoxin risk in the context of climate change necessitates a comprehensive, systems-oriented approach that links climatic variability with food safety outcomes via interconnected hazard identification, exposure assessment, and risk characterization components. This climate-smart approach will help assess how shifting climatic conditions impact fungal proliferation, mycotoxin production, dietary exposure, and the consequent risks to human and animal health.

Mycotoxin risk assessment can be conducted via two complementary approaches: the indirect diet-based approach, which combines mycotoxin occurrence data in foods with consumption information to estimate population-level exposure (Joshi et al. 2022), and the direct biomarker-based approach, which measures urinary or serum biomarkers such as AFM₁ and deoxynivalenol-glucuronide to reflect the bioavailable fraction of ingested mycotoxins (Ali et al. 2025). While indirect exposure assessment is practical, it is limited by heterogeneous contamination and dietary reporting uncertainties, whereas biomarker-based approaches better capture co-exposure despite challenges such as metabolic variability and short half-lives. Generally, mycotoxin risk assessment follows four core steps, including hazard identification, hazard characterization, exposure assessment, and risk characterization. Each step will be discussed subsequently.

### Hazard Identification

The initial phase, hazard identification, begins with the identification of mycotoxins as potential stressors, considering their specific chemical structures, toxicological properties, and potential adverse effects on human health (Dellafiora et al. 2018; Awuchi et al. 2022). This phase also encompasses an assessment of factors influencing mycotoxin production, including climatic conditions, crop storage, and processing methods (Chhaya et al., 2022). Increasing temperatures, changes in rainfall patterns, and increased humidity create favorable conditions for the proliferation of new and existing fungal spp., and their mycotoxin production capacity in agricultural commodities (Medina et al. 2017; Perrone et al. 2020). In addition, climate-induced stress on crops can increase susceptibility to infections and contamination, leading to higher levels of mycotoxins such as AFs and FBs (Perrone et al. 2020). As a result, hazards that were once geographically restricted may expand into new regions, making historical data less reliable for risk assessment.

Hazard identification in mycotoxin risk assessment depends on representative food and feed sampling together with accurate mycotoxin extraction and quantification, as risk evaluation begins by identifying the mycotoxins present and their concentrations. Because contamination is often heterogeneous and occurs in localized hotspots, inadequate sampling can lead to substantial under- or overestimation, ultimately compromising exposure assessment and risk characterization (Boshra et al. 2024). Hence, harmonized protocols from Codex Alimentarius and the European Commission ensure comparability and regulatory compliance. Following sampling, efficient extraction using solvents such as acetonitrile or methanol is required to release mycotoxins from complex matrices. Clean-up methods, including SPE, immunoaffinity columns, and QuEChERS reduce matrix effects and improve analytical sensitivity (Boshra et al. 2024).

Quantitative mycotoxin determination relies on validated analytical methods that provide accurate, precise, and reproducible concentration data for hazard classification. According to Okechukwu et al. (2023), conventional chromatographic techniques, including high-performance liquid chromatography (HPLC) coupled with ultraviolet, fluorescence, or diode-array detectors, are still used for regulated mycotoxins, often after derivatization. However, liquid chromatography–tandem mass spectrometry (LC–MS/MS) is now the gold standard for multi-mycotoxin analysis due to its high sensitivity, selectivity, and ability to quantify numerous chemically diverse toxins in a single run (Boshra et al. 2024). Also, gas chromatography–mass spectrometry (GC–MS) remains useful for volatile or semi-volatile mycotoxins and confirmatory purposes (Boshra et al. 2024), while immunochemical methods such as enzyme-linked immunosorbent assays (ELISA) and lateral flow tests provide rapid, cost-effective screening, particularly in resource-limited settings (Okechukwu et al. 2023). Recent advances, including high-resolution mass spectrometry, biosensors, and non-destructive spectroscopic tools combined with chemometric or machine-learning models, further enhance detection speed, sensitivity, and large-scale surveillance, strengthening mycotoxin risk assessment frameworks under a climate change scenario.

### Hazard characterization

Hazard characterization of mycotoxins defines the relationship between dose and adverse health outcomes by integrating toxicological evidence from experimental models and human data, and it forms the scientific basis for deriving health-based guidance values. A well-documented case is AFB₁, which has been extensively characterized as a potent genotoxic and hepatocarcinogenic compound (IARC, 1993). Long-term animal studies and epidemiological evidence from high-exposure regions have demonstrated a strong dose–response relationship between AFB₁ intake and hepatocellular carcinoma, particularly in populations with hepatitis B virus infection, leading JECFA and EFSA to conclude that no safe exposure threshold can be established (EFSA, 2020). Consequently, hazard characterization for AFB₁ is conducted using a non-threshold approach, with benchmark dose modeling (BMDL₁₀) derived for liver cancer and applied within a margin of exposure framework rather than setting a tolerable daily intake (JECFA, 2017; EFSA, 2020). In contrast, mycotoxins such as DON and ZEN exhibit threshold-dependent toxicities, including gastrointestinal disturbances and estrogenic effects, respectively. Based on dose–response data from sub-chronic and chronic studies, EFSA established group TDIs of 1 µg/kg bw/day for DON and 0.25 µg/kg bw/day for ZEN, reflecting exposure levels below which adverse effects are unlikely to occur (EFSA, 2016, 2017). These case studies illustrate how hazard characterization differentiates between genotoxic and non-genotoxic mycotoxins, identifies critical endpoints, and quantitatively defines toxicity thresholds that underpin subsequent mycotoxin risk assessment and regulatory decision-making.

Hazard characterization in the era of climate change must take into account how the inherent toxicity and health impacts of mycotoxins may change in response to changing climatic conditions. Importantly, the expression of these hazards is strongly influenced by climate variables such as temperature, humidity, and atmospheric CO₂ levels, which can modify fungal growth dynamics, infection patterns, and mycotoxin production, resulting in changes in both the occurrence and concentration of different mycotoxins in the food chain (Al-Zaban 2023; Yan et al. 2026). As a result, the severity and nature of toxicological risks may differ regionally and temporally, thus raising uncertainty in risk assessment approaches.

### Exposure assessment

Following hazard characterization, exposure assessment is conducted to estimate the extent to which individuals are exposed to different mycotoxins, especially via food consumption. This stage involves precise measurement of mycotoxin levels in affected foods, accounting for variations in consumption habits, portion sizes, and potential routes of exposure (EFSA 2020). To achieve a more accurate approximation of the mean concentrations of mycotoxins consumed through food by the general population, the EFSA has suggested using Total Diet Studies (TDSs). This approach considers the overall exposure through entire diets as consumed rather than focusing solely on the contamination levels in raw commodities. Some countries, including China, France, and South Korea, have implemented country-specific Total Diet Studies (TDSs) for mycotoxins; however, many nations have not adopted this approach, leading to challenges in conducting mycotoxin exposure assessments (Karsauliya et al. 2022). Furthermore, comparing data from national surveys proves to be challenging due to differences in the methodologies used for data collection, and not all age groups, like children and adults, are uniformly included. According to Karsauliya et al. (2022), analytical challenges further constrain exposure assessment, as the diverse physicochemical properties of mycotoxins complicate their simultaneous extraction and quantification, particularly in multi-mycotoxin analyses.

Besides consumption data and analytical instruments, climate change variables, particularly temperature, humidity, and CO2, influence exposure assessment by altering fungal infection rates and mycotoxin production capacity in the field and during storage. For example, Kimanya (2015) affirmed that high temperatures and drought stress may accelerate AF contamination in field crops, while elevated CO2 and temperature variation were confirmed to promote the biosynthesis of *Fusarium*-associated mycotoxins, such as FBs, DON, and ZEN, on the farm (Hay et al. 2021; Bakker et al. 2023). Similarly, temperatures and humidity fluctuations during storage can promote fungal growth and mycotoxin accumulation in farm products during storage, especially under conditions of inadequate drying, poor ventilation, and suboptimal storage infrastructure (Okori et al. 2022). As a result, risk assessment approaches must account for post-harvest climate sensitivity when estimating exposure under real-world conditions.

### Risk characterization

The risk characterization phase integrates data from the preceding stages (hazard identification, hazard characterization, and exposure assessment) to comprehensively evaluate mycotoxin risk, quantifying both the likelihood and severity of potential adverse health effects (Karsauliya et al. 2022). Climate change influences all three components by altering fungal distribution, increasing mycotoxin contamination levels in staple crops, and affecting food security in climate-sensitive regions. Various metrics are employed for risk characterization, including the Hazard Index (HI), Combined Margin of Exposure (MOET), Point of Departure Index (PODI), and Toxic Unit Summation (TUS), with HI and MOET being the most applied. Risk estimation can follow a deterministic approach, using single-point estimates such as mean or maximum values to calculate HQ or MOE, which is straightforward but does not account for variability or uncertainty, or a probabilistic approach, which applies statistical distributions and Monte Carlo simulations to account for population variability, co-exposure, and uncertainty, producing ranges of risk estimates and identifying high-risk subgroups (Assunção et al. 2016). The selection between HI and MOE/MOET approaches depends on whether mycotoxins exhibit threshold or non-threshold toxicological effects. HI is used for threshold mycotoxins with established health-based guidance values such as TDI, PMTDI, or Reference Dose (RfD), including DON, FBs, ZEN, and OTA (Wu et al. 2024). In contrast, MOE/MOET is applied to non-threshold, genotoxic carcinogens such as AFB_1_, where no safe exposure level can be defined (Huang et al. 2021; Joshi et al. 2022). MOET is derived from BMDL, with lower values indicating increasing public health concern.

In mycotoxin risk assessment, quantitative risk characterization relies on several toxicological and exposure metrics to evaluate potential health effects. Key parameters include the No Observed Adverse Effect Level (NOAEL) and Lowest Observed Adverse Effect Level (LOAEL), which define the exposure levels at which no or minimal adverse effects are observed in experimental studies. The Benchmark Dose (BMD) approach provides a statistically derived dose associated with a predefined increase in adverse effect incidence, often used to establish reference points for risk assessment. Health-based guidance values such as the Acceptable Daily Intake (ADI), Provisional Maximum Tolerable Daily Intake (PMTDI), or Tolerable Daily Intake (TDI) are derived from these reference points by applying appropriate safety factors (Huang et al. 2021). Exposure metrics, including Probable Daily Intake (PDI) and Estimated Daily Intake (EDI), quantify the actual intake of mycotoxins from dietary or biomarker data. These values are compared with toxicological reference points to assess potential risk and guide regulatory decisions (EFSA, 2017; Assunção et al., 2016).

Risk characterization of mycotoxins can be conducted for individual or combined mycotoxins. For individual mycotoxins, it involves integrating exposure data with toxicological reference values to estimate the potential health risks to humans or animals. For instance, Assunção et al. (2016) assessed various mycotoxins, including FBs (FB_1_ and FB_2_), TCTs (NIV, DON, and T2-Toxin), and AFs (AFB_1_, AFB_2_, AFG_1_, and AFM_1_) in breakfast cereals for children aged 0–3 years. Individual MoEs were consistently above 10,000 for all mycotoxins except AFB_1_, indicating minimal risk, but the cumulative MoET was below 10,000, highlighting a potential health concern. Several studies have also examined the risk characterization of combined mycotoxins in different foods such as nuts, sugar cane juice, rice, sesame, maize, and peanut (Cunha et al. 2018; Abdallah et al. 2020; Do et al. 2020). Pongpraket et al. (2020) analyzed ZEN, AFs, OTA, DON, T-2 toxin, FBs, and NIV in Thai black and white sesame seeds to assess health risks using margin of exposure, hazard quotient, and liver cancer risk metrics. Mycotoxins were detected in 21.5% of the 200 samples, with 19.5% containing single toxins, 2% multiple toxins, and 9% contaminated with AFs. The estimated liver cancer risk from AFB_1_ in high consumers exceeded 1 case per million, indicating potential health concerns.

Similarly, Huang et al. (2021) evaluated exposure to 23 mycotoxins in adults from the Yangtze River Delta, China, detecting eight mycotoxins in urine samples, with 22.47% containing multiple toxins, notably FB_1_, DON, ZEN, and AFB_1_. While individual risk assessments indicated potential adverse effects from AFB_1_, ZEN, FB_1_, and OTA, combined exposure to ZEN and DON suggested possible endocrine-disrupting risks based on the hazard index approach. The authors concluded that the MOE for both FB_1_ and AFB_1_ might raise potential health concerns, with AFB_1_ identified as the primary contributor. In another study, Joshi et al. (2022) examined the contamination and risk assessment of multiple mycotoxins (AFs, DON, FB_1_, OTA, ZEN, and DON) in 45 maize samples from Nepal. The most prevalent mycotoxins were DON (100%) and AFs (78%), followed by FBs and ZEN (both 76%), as well as OTA (62%). Notably, each sample contained at least two mycotoxins, whereas 87% of the samples had three or more mycotoxins. The calculated daily intake, MOE, and risk of liver cancer associated with the consumption of maize were 30.46 ng/kg bw/day, 5.58, and 0.38 cancer cases/year/100,000 population, respectively. The risk assessment suggests that maize poses a potential risk for AF exposure in Nepal. Available evidence indicates that AFB_1_ and FB_1_ pose notable human health risks, particularly under co-exposure scenarios. In contrast, mycotoxins such as ZEN and DON, although associated with adverse effects, generally present lower risks at typical dietary intakes.

As revealed by Huang et al. (2021), a significant obstacle in cumulative risk assessment is the frequent co-occurrence of mycotoxins with various toxicological endpoints, such as kidney toxicity from OTA, hepatic carcinogenicity from AFs, and endocrine disruption from ZEN, which complicates basic additive methodologies. Consequently, endpoint-based categorization by target organ, mechanism of action, or adverse outcome pathway is crucial, along with the assessment of potential additive, synergistic, or antagonistic interactions. Consequently, the HI method is more appropriate for compounds with similar toxicological endpoints or modes of action, as the concentration-addition assumption underlying HI may not adequately represent interactions among mechanistically unrelated toxins (Assunção1 et al., 2016). This review shows that climate change alters mycotoxin risk assessment, rather than merely increasing mycotoxin prevalence and levels in food and feed. Unfortunately, traditional risk assessment depends heavily on historical contamination data and single mycotoxin assessments, which may underestimate future risks, as elevated CO_2_, rising temperatures, changing rainfall, and drought may increase the likelihood of multi-mycotoxin co-exposure. Consequently, climate-responsive risk assessment should integrate TDI-based methods (HI/HQ) for threshold toxins with MOE/MOET for non-threshold carcinogenic mycotoxins to provide a more comprehensive characterization of cumulative risk. Incorporating predictive modelling and real-time environmental monitoring will further enhance exposure estimates and enable proactive food and feed safety management in the face of changing climatic conditions.

## Mitigation strategies for mycotoxin contamination in food and feed to reduce human and animal exposure

Mycotoxin contamination in food and feed presents significant health risks to humans and animals, necessitating effective mitigation strategies to reduce exposure and safeguard public health. Reducing mycotoxin levels is critical to prevent acute and chronic toxic effects, including immunosuppression, carcinogenicity, and endocrine disruption (Kościelecka et al. 2023). Mycotoxin mitigation strategies can be classified into preventive measures (including pre-harvest and post-harvest measures) and detoxification approaches, which include physical, chemical, and biological methods (Agriopoulou et al. 2020; Lalpekhlua et al. 2024). Collectively, these integrated practices are essential for mitigating mycotoxin risks under increasingly unstable climate conditions.

### Preventive strategies

**Fig. 3:**
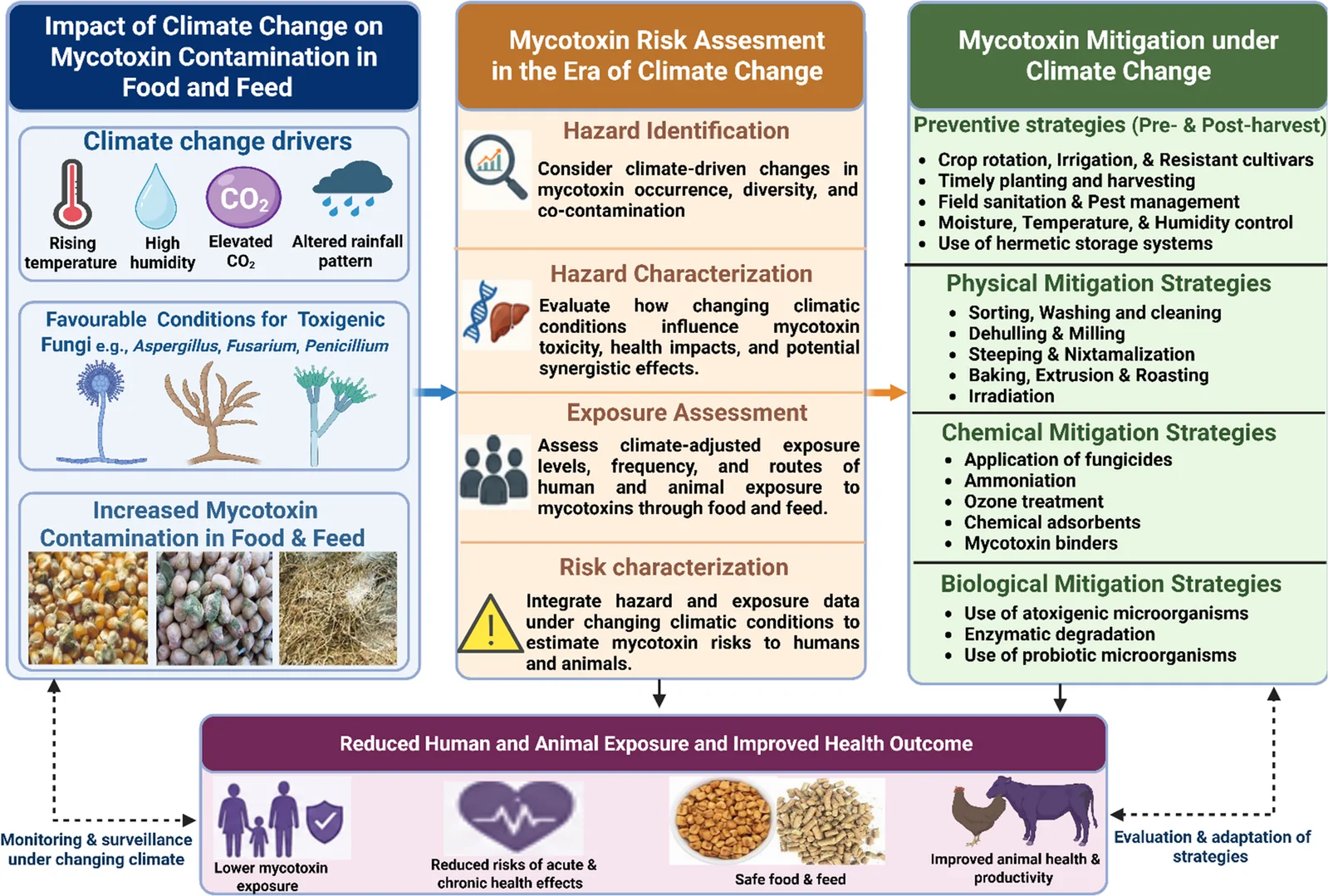
Integrated framework for mycotoxin risk assessment and mitigation under climate change

Preventive strategies are the first line of defense in mycotoxin management, focusing on minimizing contamination before it develops in the food and feed chain. These approaches target critical stages of crop production and handling where fungal growth and toxin production can be effectively controlled. By reducing environmental and agronomic risk factors, preventive measures help limit the incidence of toxigenic fungi in agricultural systems. They are broadly categorized into pre-harvest and post-harvest management practices. Together, these strategies play a central role in ensuring food safety and reducing mycotoxin-related health risks. Figure 3 illustrates the impact of climate change on mycotoxin contamination, the major stages of mycotoxin risk assessment, and mitigation strategies used to reduce human and animal exposure and improve food safety.

#### Pre-harvest preventive strategies

Pre-harvest preventive strategies aim to mitigate fungal infections and subsequent mycotoxin production in the field by reducing environmental, agronomic, and biological conditions that are conducive to toxigenic fungi. These strategies are based on Good Agricultural Practices (GAP) and involve a combination of cultural, genetic, and crop management techniques that collectively enhance plant health and resilience (Kumar et al. 2023). Key pre-harvest mycotoxin mitigation strategies include crop rotation, which interrupts the continuity of soil-borne inoculum and reduces the build-up of toxigenic fungi over successive planting seasons (Dong et al. 2023); timely planting and harvesting, which help crops avoid peak periods of high humidity, drought stress, or insect pressure that favor fungal colonization (Kochiieru et al. 2021); and the use of resistant or less susceptible cultivars, which offer intrinsic genetic protection against fungal infection and diminish toxin accumulation in grains (Bissonnette et al. 2018). Moreover, field sanitation practices, including the elimination and destruction of infected crop residues, reduce the reservoir of fungal spores capable of initiating new infections in subsequent seasons. Proper irrigation management has also been confirmed to be imperative in mycotoxin reduction in food and feed, as both water stress and excessive moisture can predispose crops to fungal invasion, particularly under climate variability with unpredictable rainfall patterns (Herrera et al. 2023; Vishwakarma et al. 2024). Furthermore, effective pest and insect management is crucial in mycotoxin control, since insect damage creates physical entry points for fungi such as Aspergillus and Fusarium, thereby facilitating infection and mycotoxin formation (Okori et al. 2022).

#### Post-harvest preventive strategies

Post-harvest preventive strategies are designed to prevent fungal colonization and further mycotoxin formation in farm products during storage, handling, and transportation, and are particularly critical in the face of climate change due to increased temperatures, CO2, and humidity fluctuations that favor post-harvest fungal proliferation. These mycotoxin control measures rely on Good Manufacturing Practices (GMP) and effective storage management systems that maintain grain quality and safety after harvest. The primary post-harvest mycotoxin mitigation methods include proper drying of crops to safe moisture levels immediately after harvest, as inadequate drying remains one of the most important drivers of post-harvest fungal growth and toxin accumulation in food and feed (Nada et al. 2022). More so, Maintenance of appropriate temperature and relative humidity conditions during storage is also essential, as elevated environmental conditions under climate change can accelerate fungal metabolism and mycotoxin biosynthesis (Al-Zaban 2023; Platzer et al. 2025). Another important post-harvest intervention is moisture control within storage systems. This approach helps prevent localized dampness and hotspots that support fungal development and mycotoxin formation in a variety of farm products (Kamel et al. 2024). As revealed by Ololi et al. (2022) and Kamel et al. (2024), the use of hermetic storage systems also reduces fungal contamination and mycotoxin production in agricultural commodities by limiting oxygen availability and insect activity. Finally, hygienic handling during transit and processing is a crucial post-harvest mycotoxin prevention approach as it reduces the entrance and dissemination of fungal spores while minimizing fungal and mycotoxin cross-contamination (Dey et al. 2022).

### Physical mitigation strategies

Physical mitigation or decontamination strategies are employed after contamination to lower mycotoxin levels in food and feed by physically removing or degrading mycotoxins during processing, thereby reducing dietary exposure and associated health risks. Sorting, one of the main physical reduction methods, removes visibly molded, damaged, or discolored kernels that typically contain significantly higher concentrations of mycotoxins (Tibola et al. 2016). Washing and cleaning also reduce fungal and mycotoxin contamination in food and feed by removing dust, spores, and surface-bound toxins loosely associated with grain surfaces (Tibola et al. 2016). Dehulling, another physical control measure, is particularly effective in reducing mycotoxin levels in cereals, as many mycotoxins tend to accumulate in the outer bran layers of cereals, and their removal can significantly reduce the overall toxin content (Odukoya et al. 2024). As revealed by Tibola et al. (2016) and Khosrokhavar et al. (2024), milling processing, notably bran separation, also enhances mycotoxin reduction in farm products by targeting the mycotoxins in discarded fractions (husk and bran), while the refined flour contains reduced mycotoxin levels. Additionally, Steeping (soaking) may facilitate limited leaching of specific mycotoxins into water, depending on solubility and grain type, making it a significant physical mycotoxin management strategy (Khosrokhavar et al. 2024). Nixtamalization, an alkaline cooking and soaking technique employed in cereal processing, has been shown to reduce mycotoxin concentrations, particularly ZEN, DON, and FBs in maize and sorghum (Odukoya et al. 2021; Maris et al., 2025). Thermal processing methods such as roasting, baking, and extrusion have been shown to reduce a variety of mycotoxins, including AFs, DON, OTA, FBs, and ZEN, in agricultural commodities, particularly cereals, through heat-induced degradation (Carbon and Lee 2022; Teixido-Orries et al. 2025; Adelusi et al. 2026). Irradiation, another important physical control technique, enhances mycotoxin reduction indirectly in food and feed by decreasing fungal load and, in some cases, modifying toxin structure, as demonstrated by Sun et al. (2022). Overall, the effectiveness of these decontamination strategies depends on several factors, including the type of mycotoxin, food matrix, and processing parameters (temperature, duration, and moisture content), but they remain valuable tools for reducing mycotoxin exposure in already contaminated agricultural commodities.

### Chemical mitigation strategies

Chemical strategies aim to prevent fungal growth or detoxify mycotoxins in crops and feed. Pre-harvest fungicide application can mitigate toxigenic fungal infestations in crops, thereby limiting the production of their attendant mycotoxins. Post-harvest chemical treatments, including ammoniation, ozone exposure, and the use of approved preservatives, reduce the mycotoxin load in contaminated grains (Conte et al. 2020; Jiménez 2020). Ammoniation, for example, has been shown to lower aflatoxin levels in maize and peanuts substantially (Chain et al. 2021). Chemical adsorbents and mycotoxin binders are widely used in feed to reduce systemic exposure to animals. These substances, including clays, activated carbon, and synthetic polymers, bind mycotoxins in the gastrointestinal tract, preventing their absorption into the bloodstream (Bočarov-Stančić et al. 2024; Puvača et al. 2023). This approach is particularly effective for AFs and OTA and is routinely incorporated into animal feed management practices to safeguard livestock health (Akinmoladun et al. 2025; Tadele et al. 2023). While chemical strategies can be highly effective, careful regulation and monitoring are required to avoid introducing residues or toxic by-products. The combination of pre-harvest fungicide application and post-harvest chemical detoxification ensures a multi-tiered approach that reduces mycotoxin prevalence and protects both human and animal health (Bereda 2025).

### Biological mitigation strategies

Biological strategies leverage naturally occurring microorganisms or enzymes to inhibit mycotoxin-producing fungi or degrade mycotoxins. Beneficial microorganisms such as *Bacillus subtilis*,* Trichoderma harzianum*, and specific lactic acid bacteria (LAB) strains have shown antifungal activity against *Aspergillus*,* Fusarium*, and *Penicillium* species (Chen et al. 2021a, 2021b; Gajbhiye and Kapadnis 2016; Hirozawa et al. 2023). This approach reduces mycotoxin contamination in crops and stored grains. Enzymatic degradation offers another layer of biological control. Specific enzymes, produced by microorganisms or added as feed supplements, can detoxify DON, T-2, HT-2, and other trichothecenes, converting them into less toxic or non-toxic metabolites (Nešić et al. 2021). These approaches are particularly valuable in feed and animal diets where exposure can otherwise lead to reduced growth, immune suppression, or mycotoxicosis in livestock (Akande et al. 2006). Probiotic microorganisms, including *Saccharomyces cerevisiae* and LAB, can be used as feed additives to bind mycotoxins in the gut, reducing systemic absorption and mitigating health risks (Zoghi et al. 2022). Integrating these biological strategies with complementary physical and chemical interventions provides a holistic framework for mycotoxin management, enhancing food and feed safety, reducing human and animal exposure, and safeguarding public and animal health.

## Conclusion and future perspective

Mycotoxins remain a significant food and feed safety challenge globally, with AFs, FBs, DON, ZEN, OTA, and TCTs most frequently contaminating staple crops and animal feeds. This review highlights that exposure in humans and animals occurs through multiple routes and that both single and co-occurring mycotoxins are associated with serious adverse health outcomes, including carcinogenic, immunotoxic, neurotoxic, and endocrine-disrupting effects. Central to managing these risks is robust mycotoxin risk assessment, which integrates hazard identification, hazard characterization, exposure assessment, and risk characterization to inform regulatory limits and control strategies. However, climate change is altering fungal ecology and exposure patterns, increasing the occurrence and co-occurrence of multiple mycotoxins, while many current risk assessment frameworks and regulations remain largely aflatoxin-centric and based on historical, single-toxin assumptions. This mismatch may lead to underestimation of current and future mycotoxin risks, particularly in regions already vulnerable to food insecurity and climate stress.

Future risk evaluations should shift from single mycotoxin approaches to climate-responsive multi-mycotoxin frameworks that account for co-exposure, mixture toxicity, emerging mycotoxins, and climate-driven changes in contamination patterns. This will require stronger surveillance systems, standardized multi-mycotoxin analytical methods, and greater integration of human and animal biomonitoring data to improve exposure assessments. Probabilistic and cumulative risk assessment models should complement traditional deterministic methods to better reflect real-world variabilities and uncertainties. Across the food and feed chains, mitigation strategies should combine good agricultural and storage practices with biological, physical, and chemical interventions supported by early warning systems and updated risk assessment data. Together, these measures will improve regulatory decision-making, strengthen the safety and resilience of food and feed systems, and ultimately reduce human and animal exposure to multiple mycotoxins in changing climates.

## Supplementary Information

Below is the link to the electronic supplementary material.

Supplementary file 1 (DOCX 32.0 KB)

## Data Availability

No datasets were generated or analysed during the current study.

## References

[CR1] Abbès S, Ben Salah-Abbès J, Jebali R, Ben Younes R, Oueslati R (2016) Interaction of aflatoxin B_1_ and fumonisin B_1_ in mice causes immunotoxicity and oxidative stress: possible protective role using lactic acid bacteria. J Immunotoxicol 13:46–54 10.3109/1547691X.2014.997905 25585958

[CR2] Abdallah MF, Audenaert K, Lust L, Landschoot S, Bekaert B, Haesaert G, De Boevre M, De Saeger S (2020) Risk characterization and quantification of mycotoxins and their producing fungi in sugarcane juice: a neglected problem in a widely consumed traditional beverage. Food Control 108:106811

[CR3] Adelusi OA, Gbashi S, Adebiyi JA, Makhuvele R, Aasa AO, Oladeji OM, Khoza M, Okoth S, Njobeh PB (2022) Seasonal diversity and occurrence of filamentous fungi in smallholder dairy cattle feeds and feedstuffs in South Africa. J Fungi 8(11):1192 10.3390/jof8111192 PMC9696519 36422014

[CR4] Adelusi OA, Ajala O, Owheruo JO, Okechukwu VO, Richard FT, Obadina AO, Adekoya IO, Kondiah K, De Saeger S, Njobeh PB (2026) Extrusion technology for mycotoxin reduction in cereals: process parameters, technological advances, advantages, limitations, and future perspectives. Appl Food Res. 10.1016/j.afres.2026.101938

[CR6] Agriopoulou S, Stamatelopoulou E, Varzakas T (2020) Advances in occurrence, importance, and mycotoxin control strategies: Prevention and detoxification in foods. Foods 9(2):137 10.3390/foods9020137 PMC7074356 32012820

[CR7] Akande K, Abubakar M, Adegbola T, Bogoro S (2006) Nutritional and health implications of mycotoxins in animal feeds: a review. Pakistan J Nutr 5(5):398–403

[CR8] Akinmoladun OF, Fon FN, Nji Q, Adeniji OO, Tangni EK, Njobeh PB (2025) Multiple mycotoxin contamination in livestock feed: Implications for animal Health, productivity, and food safety. Toxins 17(8):365 10.3390/toxins17080365 PMC12389956 40864041

[CR9] Akinrinmade FJ, Akinrinde AS, Amid A (2016) Changes in serum cytokine levels, hepatic and intestinal morphology in aflatoxin B1-induced injury: modulatory roles of melatonin and flavonoid-rich fractions from Chromolena odorata. Mycotoxin Res 32:53–60 10.1007/s12550-016-0239-9 26798045

[CR10] Al-Zaban MI (2023) Impacts of temperature and water activity interactions on growth, aflatoxin B1 production and expression of major biosynthetic genes of AFB1 in Aspergillus flavus isolates. Microorganisms 11(5):1199 10.3390/microorganisms11051199 PMC10222184 37317174

[CR12] Alassane-Kpembi I, Kolf-Clauw M, Gauthier T, Abrami R, Abiola FA, Oswald IP, Puel O (2013) New insights into mycotoxin mixtures: The toxicity of low doses of Type B trichothecenes on intestinal epithelial cells is synergistic. Toxicol Appl Pharmcol 272:191–198 10.1016/j.taap.2013.05.023 23735874

[CR13] Alassane-Kpembi I, Puel O, Oswald IP (2015) Toxicological interactions between the mycotoxins deoxynivalenol, nivalenol and their acetylated derivatives in intestinal epithelial cells. Arch Toxicol 89:1337–1346 10.1007/s00204-014-1309-4 25033990

[CR14] Ali N (2019) Aflatoxins in rice: Worldwide occurrence and public health perspectives. Toxicol Rep 6:1188–1197 10.1016/j.toxrep.2019.11.007 PMC6872864 31768330

[CR15] Ali S, Franco BB, Rezende VT, Ullah S, Wahab N, Freire LGD, Oliveira CAF (2025) Children’s exposure to mycotoxins and risk characterization using urinary biomarkers in São Paulo, Brazil. Environ Pollut, 385:127160 10.1016/j.envpol.2025.127160 40998143

[CR16] Alizadeh AM, Roshandel G, Roudbarmohammadi S, Roudbary M, Sohanaki H, Ghiasian SA, Taherkhani A, Semnani S, Aghasi M (2012) Fumonisin B_1_ contamination of cereals and risk of esophageal cancer in a high-risk area in northeastern Iran. Asian Pac J Cancer Prev 13(6):2625–2628 10.7314/apjcp.2012.13.6.2625 22938431

[CR17] Altafini A, Roncada P, Guerrini A, Minkoumba Sonfack G, Fedrizzi G, Caprai E (2021) Occurrence of ochratoxin A in different types of cheese offered for sale in Italy. Toxins 13(8):540 10.3390/toxins13080540 PMC8402398 34437411

[CR18] Alvito P, Assunção RM, Bajard L, Martins C, Mengelers MJ, Mol H, Namorado S, van den Brand AD, Vasco E, Viegas S, Silva MJ (2022) Current Advances, Research Needs and Gaps in Mycotoxins Biomonitoring under the HBM4EU—Lessons Learned and Future Trends. Toxins 14(12):826 10.3390/toxins14120826 PMC9783896 36548723

[CR19] Ankwasa EM, Francis I, Ahmad T (2021) Update on mycotoxin contamination of maize and peanuts in East African Community Countries. J Food Sci Nutr Therapy 7:1–10

[CR20] Annunziata L, Campana G, De Massis MR, Aloia R, Scortichini G, Visciano P (2025) Ochratoxin A in foods and beverages: dietary exposure and risk assessment. Exposure Health 17(2):481–494

[CR24] Arnot LF, Duncan NM, Coetzer H, Botha CJ (2012) An outbreak of canine aflatoxicosis in Gauteng Province, South Africa. J S Afr Vet Assoc 83(1):1–4 10.4102/jsava.v83i1.2 23327140

[CR25] Aroud HI, May B, Dietrich H, Schweiggert R, Kemmlein S (2021) Influence of processing steps on the fate of ochratoxin A, patulin, and alternariol during production of cloudy and clear apple juices. Mycotoxin Res 37(4):341–354 10.1007/s12550-021-00443-x PMC8571144 34693499

[CR26] Assunção R, Silva MJ, Alvito P (2016) Challenges in risk assessment of multiple mycotoxins in food. World Mycotoxin J 9(5):791–811

[CR27] Awad WA, Hess M, Twarużek M, Grajewski J, Kosicki R, Böhm J, Zentek J (2011) The Impact of the *Fusarium* mycotoxin deoxynivalenol on the health and performance of broiler chickens. Int J Mol Sci 12(11):7996–8012 10.3390/ijms12117996 PMC3233452 22174646

[CR28] Awapak D, Petchkongkaew A, Sulyok M, Krska R (2021) Co-occurrence and toxicological relevance of secondary metabolites in dairy cow feed from Thailand. Food Additives & Contaminants: Part A 38(6):1013–1027 10.1080/19440049.2021.1905186 33861173

[CR30] Awuchi CG, Ondari EN, Nwozo S, Odongo GA, Eseoghene IJ, Twinomuhwezi H, Ogbonna CU, Upadhyay AK, Adeleye AO, Okpala COR (2022) Mycotoxins’ toxicological mechanisms involving humans, livestock and their associated health concerns: a review. Toxins 14:167 10.3390/toxins14030167 PMC8949390 35324664

[CR31] Bakker MG, Whitaker BK, McCormick SP, Ainsworth EA, Vaughan MM (2023) Manipulating atmospheric CO2 concentration induces shifts in wheat leaf and spike microbiomes and in *Fusarium* pathogen communities. Front Microbiol 14:1271219 10.3389/fmicb.2023.1271219 PMC10595150 37881249

[CR32] Bastianello SS, Nesbit JW, Lange AL (1987) Pathological findings in a natural outbreak of aflatoxicosis in dogs. Onderstepoort J Vet Res 54(4):635–640 3444619

[CR34] Bereda G (2025) Toxicological Impacts, Mitigation, and Policy Strategies of Food Contaminants. A Global Perspective and Comprehensive Narrative Review 10.1016/j.crtox.2025.100268 PMC12664818 41328358

[CR35] Bereka TY, Kuyu CG, Tolera KD, Addis EM (2022) Current postharvest practices and aflatoxin contamination awareness amongst maize producers in Jimma Zone, Southwest of Ethiopia. World Mycotoxin J 15(1):35–43

[CR36] Bianco G, Fontanella B, Severino L, Quaroni A, Autore G, Marzocco S (2012) Nivalenol and deoxynivalenol affect rat intestinal epithelial cells: a concentration related study. PLoS One 7:e52051 10.1371/journal.pone.0052051 PMC3522672 23251682

[CR317] Binder EM, Tan LM, Chin LJ, Handl J, Richard J (2007) Worldwide occurrence of mycotoxins in commodities, feeds and feed ingredients. Feed Sci Technol 137(3-4):265-82

[CR37] BIOMIN World Mycotoxin Survey. 2020. BIOMIN Mycotoxin Survey Q3 2020 https://www.biomin.net/science-hub/biomin-mycotoxin-survey-q3-2020-results

[CR38] Bissonnette KM, Kolb FL, Ames KA, Bradley CA (2018) Effect of wheat cultivar on the concentration of Fusarium mycotoxins in wheat stems. Plant Dis 102(12):2539–2544 10.1094/PDIS-12-17-2034-RE 30252626

[CR39] Bočarov-Stančić A, Lopičić Z, Krstović S, Krulj J, Milojković J, Maslovarić M, Bodroža-Solarov M (2024) Agents of different origins for reduction of mycotoxins’ level in feed. Ann Anim Sci 24(3):707–729

[CR40] Bogueva D, McClements DJ (2023) Safety and nutritional risks associated with plant-based meat alternatives. Sustainability 15(19):14336

[CR41] Boshra MH, El-Housseiny GS, Farag MM, Aboshanab KM (2024) Innovative approaches for mycotoxin detection in various food categories. AMB Express 14(1):7 10.1186/s13568-024-01662-y PMC10786816 38216801

[CR42] Budagova G, Buyukunal SK, Muratoglu K (2025) Determination of mycotoxins in breakfast cereals by LC-MS/MS. Food Control 168:110971

[CR43] Burel C, Tanguy M, Guerre P, Boilletot E, Cariolet R, Queguiner M, Fravalo P (2013) Effect of low dose of fumonisins on pig health: immune status, intestinal microbiota and sensitivity to *Salmonella*. Toxins (Basel) 5(4):841–864 10.3390/toxins5040841 PMC3705294 23612754

[CR44] Cantero-Gómez M, López-Puertollano D, Navarro-Fuertes I, Abad-Somovilla A, Salcedo-Domínguez J, González-Candelas L, Ballester AR, Abad-Fuentes A, Mercader JV (2026) Validation of novel immunoassays according to European legislation for the rapid analysis of ochratoxin A in cereal, grape, and dried vine fruit samples. J Food Compos Anal 149:108569

[CR45] Carbon HN, Lee HJ (2022) Varied reduction of ochratoxin A in brown and white rice during roasting. World Mycotoxin J 15(4):361–368

[CR46] Castell A, Arroyo-Manzanares N, Campillo N, Sanz-Fernández S, Rodríguez-Estévez V, Roquet J, González A, Fenoll J, Viñas P (2025) Reliable and sensitive analytical platform to assess dietary exposure of pigs to mycotoxins and explore potential urinary biomarkers. Talanta 286:127441 10.1016/j.talanta.2024.127441 39733520

[CR47] Castells M, Marin S, Sanchis V, Ramos AJ (2019) Mycotoxins in cereals and cereal products: a review on occurrence and recent methods of analysis. Trends Food Sci Technol 88:111–124

[CR48] Casu A, Camardo Leggieri M, Toscano P, Battilani P (2024) Changing climate, shifting mycotoxins: a comprehensive review of climate change impact on mycotoxin contamination. Compr Rev Food Sci Food Saf 23(2):e13323 10.1111/1541-4337.13323 38477222

[CR49] Chain EP o. C. i. t., Schrenk F, Bignami D, Bodin M, Chipman L, del Mazo JK, Grasl-Kraupp J, Hoogenboom B, Leblanc L, J. C. and, Nebbia CS (2021) Assessment of an application on a detoxification process of groundnut press cake for aflatoxins by ammoniation. EFSA Journal, 19(12), e07035 10.2903/j.efsa.2021.7035 PMC8690986 34976165

[CR50] Chalyy ZA, Kiseleva MG, Sedova IB, Minaeva LP, Sheveleva SA, Tutelyan VA (2021) Dried fruits marketed in Russia: multi-mycotoxin contamination. Vopr Pitan 90(1):33–39 10.33029/0042-8833-2021-90-1-33-39 33740325

[CR51] Changwa R, De Boevre M, De Saeger S, Njobeh PB (2021) Feed-based multi-mycotoxin occurrence in smallholder dairy farming systems of South Africa: the case of Limpopo and Free State. Toxins (Basel) 13(2):166 10.3390/toxins13020166 PMC7927053 33671584

[CR53] Chen P, Liu T, Jiang S, Yang Z, Huang L, Liu F (2017) Effects of purified zearalenone on selected immunological and histopathologic measurements of spleen in post-weanling gilts. Anim Nutr 3(3):212–218 10.1016/j.aninu.2017.04.008 PMC5941232 29767107

[CR54] Chen C, Mitchell NJ, Gratz J, Houpt ER, Gong Y, Egner PA, Groopman JD, Riley RT, Showker JL, Svensen E, Mduma ER (2018) Exposure to aflatoxin and fumonisin in children at risk for growth impairment in rural Tanzania. Environ Int 115:29–37 10.1016/j.envint.2018.03.001 PMC5989662 29544138

[CR55] Chen L, Guo W, Zheng Y, Zhou J, Liu T, Chen W, Zhang J (2020) Occurrence and characterization of fungi and mycotoxins in contaminated medicinal herbs. Toxins 12(1):30 10.3390/toxins12010030 PMC7020482 31947869

[CR56] Chen H, Ju H, Wang Y, Du G, Yan X, Cui Y, Yuan Y, Yue T (2021a) Antifungal activity and mode of action of lactic acid bacteria isolated from kefir against Penicillium expansum. Food Control 130:108274

[CR57] Chen J, Wen J, Tang Y, Shi J, Mu G, Yan R, Cai J, Long M (2021b) Research progress on fumonisin B_1_ contamination and toxicity: A Review. Molecules 26(17):5238 10.3390/molecules26175238 PMC8434385 34500671

[CR58] Chen J, Chen Y, Zhu Q, Wan J (2023) Ochratoxin A contamination and related high-yield toxin strains in Guizhou dried red chilies. Food Control 145:109438

[CR318] Chhaya RS, O'Brien J, Cummins E (2022). Feed to fork risk assessment of mycotoxins under climate change influences-recent developments 126:126-41.

[CR59] Conte G, Fontanelli M, Galli F, Cotrozzi L, Pagni L, Pellegrini E (2020) Mycotoxins in feed and food and the role of ozone in their detoxification and degradation: an update. Toxins 12(8):486 10.3390/toxins12080486 PMC7472270 32751684

[CR60] Cunha SC, Sa SV, Fernandes JO (2018) Multiple mycotoxin analysis in nut products: Occurrence and risk characterization. Food Chem Toxicol 114:260–269 10.1016/j.fct.2018.02.039 29458161

[CR61] da Rocha MEB, Freire FDCO, Maia FEF, Guedes MIF, Rondina D (2014) Mycotoxins and their effects on human and animal health. Food Control 36(1):159–165

[CR62] Dai C, Xiao X, Sun F, Zhang Y, Hoyer D, Shen J, Tang S, Velkov T (2019) T-2 toxin neurotoxicity: role of oxidative stress and mitochondrial dysfunction. Arch Toxicol 93:1–16 10.1007/s00204-019-02577-5 31570981

[CR63] Daou R, Hoteit M, Bookari K, Joubrane K, Khabbaz LR, Ismail A, Maroun RG, Khoury AE (2023) Public health risk associated with the co-occurrence of aflatoxin B_1_ and ochratoxin A in spices, herbs, and nuts in Lebanon. Front Public Health 10:1072727 10.3389/fpubh.2022.1072727 PMC9868821 36699892

[CR64] De Jesus CL, Bartley A, Welch AZ, Berry JP (2017) High incidence and levels of ochratoxin A in wines sourced from the United States. Toxins 10(1):1 10.3390/toxins10010001 PMC5793088 29267200

[CR65] de Sá SV, Monteiro CS, Fernandes JO, Pinto E, Faria MA, Cunha SC (2024) Evaluating the human neurotoxicity and toxicological interactions impact of co-occurring regulated and emerging mycotoxins. Food Res Int 184:114239 10.1016/j.foodres.2024.114239 38609220

[CR66] Dellafiora L, Dall’asta C, Galaverna G (2018) Toxicodynamics of mycotoxins in the framework of food risk assessment—An in silico perspective. Toxins 10:52 10.3390/toxins10020052 PMC5848153 29360783

[CR67] Dey DK, Kang JI, Bajpai VK, Kim K, Lee H, Sonwal S, Simal-Gandara J, Xiao J, Ali S, Huh YS, Han YK (2022) Mycotoxins in food and feed: Toxicity, preventive challenges, and advanced detection techniques for associated diseases. Crit Rev Food Sci Nutr 63: 1–22 10.1080/10408398.2022.2059650 35445609

[CR68] Do TH, Tran SC, Le CD, Nguyen HBT, Le PTT, Le HHT, Le TD, Thai-Nguyen HT (2020) Dietary exposure and health risk characterization of aflatoxin B1, ochratoxin A, fumonisin B1, and zearalenone in food from different provinces in Northern Vietnam. Food Control 112:107108

[CR69] Dong F, Chen X, Lei X, Wu D, Zhang Y, Lee YW, Mokoena MP, Olaniran AO, Li Y, Shen G, Liu X (2023) Effect of crop rotation on Fusarium mycotoxins and Fusarium species in cereals in Sichuan Province (China). Plant Dis 107(4):1060–1066 10.1094/PDIS-01-22-0024-RE 36122196

[CR71] Duarte SC, Lino CM, Pena A (2010) Mycotoxin food and feed regulation and the specific case of ochratoxin A: a review of the worldwide status. Food Addit Contam 27(10):1440–1450 10.1080/19440049.2010.497166 20658402

[CR23] EFSA, Arcella D, Gergelova P, Innocenti ML, Steinkellner H (2017) Human and animal dietary exposure to T-2 and HT‐2 toxin. EFSA J 15(8):04972 10.2903/j.efsa.2017.4972 PMC7010047 32625633

[CR72] EFSA (2011) Scientific opinion on the risks for public health related to the presence of zearalenone in food by EFSA panel on contaminants in the food chain. EFSA J 9(2):2197

[CR319] EFSA Panel on Contaminants in the Food Chain (CONTAM) (2016). Appropriateness to set a group health‐based guidance value for zearalenone and its modified forms. EFSA Journal, 14(4): e04425

[CR73] EFSA Panel on Contaminants in the Food Chain (CONTAM), Schrenk D, Bignami M, Bodin L, Chipman JK, del Mazo J, Grasl-Kraupp B, Hogstrand C, Hoogenboom L, Leblanc JC, Nebbia CS (2020) Risk assessment of aflatoxins in food. EFSA J 18(3):06040 10.2903/j.efsa.2020.6040 PMC7447885 32874256

[CR74] Ekwomadu TI, Akinola SA, Mwanza M (2021) *Fusarium* mycotoxins, their metabolites (free, emerging, and masked), food safety concerns, and health impacts. Int J Environ Res Public Health 18:11741 10.3390/ijerph182211741 PMC8618243 34831498

[CR75] El Darra N, Gambacorta L, Solfrizzo M (2019) Multi-mycotoxins occurrence in spices and herbs commercialized in Lebanon. Food Control 95:63–70

[CR76] El-Sayed RA, Jebur AB, Kang W, El-Demerdash FM (2022) An overview on the major mycotoxins in food products: Characteristics, toxicity, and analysis. J Future Foods 2(2):91–102

[CR77] Eskola M, Kos G, Elliott CT, Hajšlová J, Mayar S, Krska R (2020) Worldwide contamination of food-crops with mycotoxins: Validity of the widely cited ‘FAO estimate’of 25%. Crit Rev Food Sci Nutr 60(16):2773–2789 10.1080/10408398.2019.1658570 31478403

[CR78] European Commission (2023) Commission Regulation (EU) 2023/915 of 25 April 2023 on maximum levels for certain contaminants in food and repealing Regulation (EC) 1881/2006. Official J Eur Union L 119:103–157. https://eur-lex.europa.eu/eli/reg/2023/915/oj (accessed on 26 May 2026)

[CR79] Falkauskas R, Bakutis B, Jovaišienė J, Vaičiulienė G, Gerulis G, Kerzienė S, Jacevičienė I, Jacevičius E, Baliukonienė V (2022) Zearalenone and its metabolites in blood serum, urine, and milk of dairy cows. Animals 12(13):1651 10.3390/ani12131651 PMC9264949 35804550

[CR80] Fan K, Xu J, Jiang K, Liu X, Meng J, Di Mavungu JD, Guo W, Zhang Z, Jing J, Li H, Yao B (2019) Determination of multiple mycotoxins in paired plasma and urine samples to assess human exposure in Nanjing, China. Environ Pollut 248:865–873 10.1016/j.envpol.2019.02.091 30856502

[CR82] Ferreras MC, Benavides J, García-Pariente C, Delgado L, Fuertes M, Muñoz M, García-Marín JF, Pérez V (2013) Acute and chronic disease associated with naturally occurring T-2 mycotoxicosis in sheep. J Comp Pathol 148(2–3):236–242 10.1016/j.jcpa.2012.05.016 22819015

[CR84] Ficheux AS, Sibiril Y, Parent-Massin D (2012b) Co-exposure of *Fusarium* mycotoxins: *in vitro* myelotoxicity assessment on human hematopoietic progenitors. Toxicon 60:1171–1179 10.1016/j.toxicon.2012.08.001 22921581

[CR83] Ficheux AS, Sibiril Y, Parent-Massin D (2012a) Co-exposure of *Fusarium* mycotoxins: Invitro myelotoxicity assessment on human hematopoietic progenitors. Toxicon 60(6):1171–1179 10.1016/j.toxicon.2012.08.001 22921581

[CR85] Filali A, Betbeder AM, Baudrimont I, Benayada A, Soulaymani R, Creppy EE (2002) Ochratoxin A in human plasma in Morocco: a preliminary survey. Hum Exp Toxicol 21(5):241–245 10.1191/0960327102ht249oa 12141394

[CR86] Fink-Grernmels J (1999) Mycotoxins: their implications for human and animal health. Veterinary Q 21(4):115–120 10.1080/01652176.1999.9695005 10568000

[CR87] Franco LT, Petta T, Rottinghaus GE, Bordin K, Gomes GA, Oliveira CA (2019) Co-occurrence of mycotoxins in maize food and maize-based feed from small-scale farms in Brazil: A pilot study. Mycotoxin Res 35(1):65–73 10.1007/s12550-018-0331-4 30242616

[CR88] Freire FD, da Rocha ME (2017) Impact of mycotoxins on human health. In: Mérillon JM, Ramawat K (eds) Fungal Metabolites. Springer, Cham, Switzerland, pp 239–261

[CR89] Fu Y, Jin Y, Tian Y, Yu H, Wang R, Qi H, Feng B, Zhang J (2022) Zearalenone promotes LPS-induced oxidative stress, endoplasmic reticulum stress, and accelerates bovine mammary epithelial cell apoptosis. Int J Mol Sci 23(18):10925 10.3390/ijms231810925 PMC9500836 36142835

[CR90] Gab-Allah MA, Mekete KG, Choi K, Kim B (2021) Occurrence of major type-B trichothecenes and deoxynivalenol-3-glucoside in cereal-based products from Korea. J Food Compos Anal 99:103851

[CR92] Gajbhiye MH, Kapadnis BP (2016) Antifungal-activity-producing lactic acid bacteria as biocontrol agents in plants. Biocontrol Sci Technol 26(11):1451–1470

[CR93] Ganesan AR, Mohan K, Karthickrajan D, Pillay AA, Palanisami T, Sathishkumar P, Conterno L (2021) Distribution, toxicity, interactive effects, and detection of ochratoxin and deoxynivalenol in food: a review. Food Chem 378:131978 10.1016/j.foodchem.2021.131978 35033712

[CR94] Gao YN, Wang JQ, Li SL, Zhang YD, Zheng N (2016) Aflatoxin M_1_ cytotoxicity against human intestinal Caco-2 cells is enhanced in the presence of other mycotoxins. Food Chem Toxicol 96:79–89 10.1016/j.fct.2016.07.019 27470613

[CR95] Gao Z, Luo K, Peng J, Zhu Q, Liu C, Wang X, Li S, Zhang H (2023) The natural occurrence, toxicity mechanisms and management strategies of fumonisin B1: a review. Environ Pollut. 10.1016/j.envpol.2023.121065 36639041

[CR96] Gbashi S, Madala NE, De Saeger S, De Boevre M, Adekoya I, Adebo OA, Njobeh PB (2018) The Socio-economic impact of mycotoxin contamination in Africa. In: Njobeh PB (ed) Fungi and mycotoxins - their occurrence, impact on health and the economy as well as pre- and postharvest management strategies. InTech, Rijeka, Croatia, pp 1–20

[CR97] Gbashi S, Adelusi OA, Njobeh PB (2024) Insights from modelling sixteen years of climatic and fumonisin patterns in maize in South Africa. Sci Rep 14(1):11643. 10.1038/s41598-024-60904-y PMC11109125 38773169

[CR99] Ghafarifarsani H, Imani A, Niewold TA, Pietsch-Schmied C, Moghanlou KS (2021) Synergistic toxicity of dietary aflatoxin B1 (AFB1) and zearalenone (ZEN) in rainbow trout (*Oncorhynchus mykiss*) is attenuated by anabolic effects. Aquaculture 541:736793

[CR100] Gil-Serna J, Patiño B, Cortes L, Gonzalez-Jaen MT, Vazquez C (2015) *Aspergillus steynii* and *Aspergillus westerdijkiae* as potential risk of OTA contamination in food products in warm climates. Food Microbiol 46:168–175 10.1016/j.fm.2014.07.013 25475281

[CR101] Gong YY, Cardwell K, Hounsa A, Egal S, Turner PC, Hall AJ, Wild CP (2002) Dietary aflatoxin exposure and impaired growth in young children from Benin and Togo: cross sectional study. BMJ 325(7354):20–21 10.1136/bmj.325.7354.20 PMC116667 12098724

[CR102] Gordon MJ, Daud N, Cantlay L, Gratz SW (2026) Fusarium Mycotoxins Are Frequently Detected in Oat Grains and Oat Foods and T-2, HT-2 and HT-2-Glucoside Are Highly Bioaccessible in Oat Porridge. Toxins 18(7):295 10.3390/toxins18070295 PMC13417095 42506715

[CR103] Gruber-Dorninger C, Jenkins T, Schatzmayr G (2019) Global mycotoxin occurrence in feed: A ten-year survey. Toxins 11(7):375 10.3390/toxins11070375 PMC6669473 31252650

[CR106] Hassan HF, Abou Ghaida A, Charara A, Dimassi H, Faour H, Nahouli R, Karam L, Alwan N (2022) Exposure to ochratoxin A from rice consumption in Lebanon and United Arab Emirates: A comparative study. Int J Environ Res Public Health 19(17):11074 10.3390/ijerph191711074 PMC9518451 36078789

[CR107] Hay WT, McCormick SP, Vaughan MM (2021) Effects of atmospheric CO2 and temperature on wheat and corn susceptibility to *Fusarium graminearum* and deoxynivalenol contamination. Plants (Basel) 10(12):2582 10.3390/plants10122582 PMC8709488 34961056

[CR108] Herrera M, Cavero J, Franco-Luesma S, Álvaro-Fuentes J, Ariño A, Lorán S (2023) Mycotoxins and crop yield in maize as affected by irrigation management and tillage practices. Agronomy (Basel) 13(3):798

[CR109] Heussner AH, Bingle LEH (2015) Comparative ochratoxin toxicity: A review of the available data. Toxins 7:4253–4282 10.3390/toxins7104253 PMC4626733 26506387

[CR110] Hirozawa MT, Ono MA, Suguiura I, Bordini JG, Ono EYS (2023) Lactic acid bacteria and *Bacillus* spp. as fungal biological control agents. J Appl Microbiol 134(2):lxac083 10.1093/jambio/lxac083 36724273

[CR111] Houbraken J, Sándor Kocsubé CM, Visagie N, Yilmaz X-C, Wang M, Meijer B, Kraak et al (2020) Classification of Aspergillus, Penicillium, Talaromyces and related genera (Eurotiales): An overview of families, genera, subgenera, sections, series and species. Stud Mycol 9:5–169 10.1016/j.simyco.2020.05.002 PMC7426331 32855739

[CR112] Hsia CC, Wu ZY, Li YS, Zhang F, Sun ZT (2004) Nivalenol, a main *Fusarium* toxin in dietary foods from high-risk areas of cancer of esophagus and gastric cardia in China, induced benign and malignant tumors in mice. Oncol Rep 12:449–456 15254715

[CR113] Huang S, Zheng N, Fan C, Cheng M, Wang S, Jabar A, Wang J, Cheng J (2018) Effects of aflatoxin B_1_ combined with ochratoxin A and/or zearalenone on metabolism, immune function, and antioxidant status in lactating dairy goats. Asian-Australasian J Anim Sci 31(4):505–513 10.5713/ajas.17.0279 PMC5838322 28920416

[CR114] Huang Q, Jiang K, Tang Z, Fan K, Meng J, Nie D, Zhao Z, Wu Y, Han Z (2021) Exposure assessment of multiple mycotoxins and cumulative health risk assessment: A biomonitoring-based Study in the Yangtze River Delta, China. Toxins 13(2):103 10.3390/toxins13020103 PMC7912756 33535530

[CR115] Hughes DM, Gahl MJ, Graham CH, Grieb SL (1999) Overt signs of toxicity to dogs and cats of dietary deoxynivalenol. J Anim Sci 77(3):693–700 10.2527/1999.773693x 10229366

[CR116] International Agency for Research on Cancer (1993) Monographs on the Evaluation of Carcinogenic Risks to Humans. 56, 599

[CR118] Iqbal SZ (2021) Mycotoxins in food, recent development in food analysis and future challenges; a review. Curr Opin Food Sci 42:237–247

[CR119] Iqbal SZ, Nisar S, Asi MR, Jinap S (2014) Natural incidence of aflatoxins, ochratoxin A and zearalenone in chicken meat and eggs. Food Control 43:98–103

[CR120] Janik E, Niemcewicz M, Podogrocki M, Ceremuga M, Stela M, Bijak M (2021) T-2 Toxin—the most toxic trichothecene mycotoxin: metabolism, toxicity, and decontamination strategies. Molecules 26(22):6868 10.3390/molecules26226868 PMC8618548 34833960

[CR121] Jedidi I, Mateo EM, Marín P, Jiménez M, Said S, González-Jaén MT (2021) Contamination of wheat, barley, and maize seeds with toxigenic *Fusarium* species and their mycotoxins in Tunisia. J AOAC Int 104(4):959–967 10.1093/jaoacint/qsab020 33576795

[CR122] Ji J, Zhu P, Cui F, Pi F, Zhang Y, Li Y, Wang J, Sun X (2017) The antagonistic effect of mycotoxins deoxynivalenol and zearalenone on metabolic profiling in serum and liver of mice. Toxins, 9(1), 28 10.3390/toxins9010028 PMC5308260 28075412

[CR123] Ji J, Wang Q, Wu H, Xia S, Guo H, Blaženović I, Sun X (2019) Insights into cellular metabolic pathways of the combined toxicity responses of Caco-2 cells exposed to deoxynivalenol, zearalenone and Aflatoxin B1. Food Chem Toxicol 126:106–112 10.1016/j.fct.2018.12.052 30668976

[CR124] Jia R, Ma Q, Fan Y, Ji C, Zhang J, Liu T, Zhao L (2016) The toxic effects of combined aflatoxins and zearalenone in naturally contaminated diets on laying performance, egg quality and mycotoxins residues in eggs of layers and the protective effect of Bacillus subtilis biodegradation product. Food Chem Toxicol 90:142–150 10.1016/j.fct.2016.02.010 26891816

[CR125] Jia B, Lin H, Yu S, Liu N, Yu D, Wu A (2023) Mycotoxin deoxynivalenol-induced intestinal flora disorders, dysfunction and organ damage in broilers and pigs. J Hazard Mater 451:131172 10.1016/j.jhazmat.2023.131172 36907058

[CR126] Jiang SZ, Yang ZB, Yang WR, Gao J, Liu FX, Chen CC, Chi F (2010) Physiopathological effects of zearalenone in post-weaning female piglets with or without montmorillonite clay adsorbent. Livest Sci 131:130–136

[CR127] Jiang Y, Hansen PJ, Xiao Y, Amaral TF, Vyas D, Adesogan AT (2019) Aflatoxin compromises development of the preimplantation bovine embryo through mechanisms independent of reactive oxygen production. J Dairy Sci 102(11):10506–10513 10.3168/jds.2019-16839 31521360

[CR128] Jiménez JRM (2020) Assessment of Grain Safety in Developing Nations. The University of Nebraska-Lincoln

[CR129] Jorquera-Pereira D, Pavón-Pérez J, Ríos-Gajardo G (2021) Identification of type B trichothecenes and zearalenone in Chilean cereals by planar chromatography coupled to mass spectroscopy. Food Addit Contam Part A Chem Anal Control Expo Risk Assess 38(10):1778–1787 10.1080/19440049.2021.1948618 34254899

[CR130] Joshi P, Chauysrinule C, Mahakarnchanakul W, Maneeboon T (2022) Multi-mycotoxin contamination, mold incidence and risk assessment of aflatoxin in maize kernels originating from Nepal. Microbiol Res 13(2):258–277

[CR131] Kamala A, Shirima C, Jani B, Bakari M, Sillo H, Rusibamayila N, De Saeger S, Kimanya M, Gong Y, Simba A (2018) Outbreak of an acute aflatoxicosis in Tanzania during 2016. World Mycotoxin J 11:311–320

[CR132] Kamel RM, Assaha DV, El-kholy M, Elsawy HI (2024) Influence of maize (Zea mays L.) moisture content stored in various compacted multi-layer hermetic bags on seed quality and mycotoxins contamination. J Stored Prod Res 107:102319

[CR134] Kang’Ethe EK, Sirma AJ, Murithi G, Mburugu-Mosoti CK, Ouko EO, Korhonen HJ, Nduhiu GJ, Mungatu JK, Joutsjoki V, Lindfors E, Ramo S (2017) Occurrence of mycotoxins in food, feed, and milk in two counties from different agro-ecological zones and with historical outbreak of aflatoxins and fumonisins poisonings in Kenya. Food Qual Saf 1(3):161–170

[CR135] Kara GN, Ozbey F, Kabak B (2015) Co-occurrence of aflatoxins and ochratoxin A in cereal flours commercialised in Turkey. Food Control 54:275–281

[CR136] Karsauliya K, Yahavi C, Pandey A, Bhateria M, Sonker AK, Pandey H, Sharma M, Singh SP (2022) Co-occurrence of mycotoxins: A review on bioanalytical methods for simultaneous analysis in human biological samples, mixture toxicity and risk assessment strategies. Toxicon 218:25–39 10.1016/j.toxicon.2022.08.016 36049662

[CR137] Keck BB, Bodine AB (2006) The effects of fumonisin B_1_ on viability and mitogenic response of avian immune cells. Poult Sci 85:1020–1024 10.1093/ps/85.6.1020 16776470

[CR139] Khan RB, Phulukdaree A, Chuturgoon AA (2018) Fumonisin B_1_ induces oxidative stress in oesophageal (SNO) cancer cells. Toxicon 141:104–111 10.1016/j.toxicon.2017.12.041 29233736

[CR140] Khosrokhavar R, Ershadi A, Jafari-Asl M, Gholami R, Aliabadi S, Yazdi F, Mousavi Khaneghah A (2024) Mycotoxin mitigation by combined dry grinding before corn wet milling and steeping procedures. Int J Environ Anal Chem 104(15):3548–3565

[CR141] Kifer D, Jakšić D, Šegvić Klarić M (2020) Assessing the effect of mycotoxin combinations: which mathematical model is (the most) appropriate? Toxins 2(3):153 10.3390/toxins12030153 PMC7150917 32121330

[CR142] Kimanya ME (2015) The health impacts of mycotoxins in the eastern Africa region. Curr Opin Food Sci 6:7–11

[CR144] Knutsen HK, Alexander J, Barregård L, Bignami M, Brüschweiler B, Ceccatelli S, Cottrill B, Dinovi M, Edler L, Grasl-Kraupp B (2017) Risks for animal health related to the presence of zearalenone and its modified forms in feed. EFSA J Eur Food Saf Auth 15(7):e04851. 10.2903/j.efsa.2017.4851 PMC7009830 32625539

[CR145] Kochiieru Y, Mankevičienė A, Cesevičienė J, Semaškienė R, Ramanauskienė J, Gorash A, Venslovas E (2021) The impact of harvesting time on Fusarium mycotoxins in spring wheat grain and their interaction with grain quality. Agronomy 11(4):642

[CR146] Komsky-Elbaz A, Saktsier M, Roth Z (2018) Aflatoxin B_1_ impairs sperm quality and fertilization competence. Toxicology 393:42–50 10.1016/j.tox.2017.11.007 29113834

[CR148] Kościelecka K, Kuć A, Kubik-Machura D, Męcik-Kronenberg T, Włodarek J, Radko L (2023) Endocrine effect of some mycotoxins on humans: a clinical review of the ways to mitigate the action of mycotoxins. Toxins 15(9):515 10.3390/toxins15090515 PMC10535190 37755941

[CR147] Kos J, Anić M, Radić B, Zadravec M, Janić Hajnal E, Pleadin J (2023) Climate change—a global threat resulting in increasing mycotoxin occurrence. Foods 12(14):2704 10.3390/foods12142704 PMC10379110 37509796

[CR149] Kosicki R, Twarużek M, Dopierała P, Rudzki B, Grajewski J (2020) Occurrence of mycotoxins in winter rye varieties cultivated in Poland (2017–2019). Toxins (Basel) 12(6):423 10.3390/toxins12060423 PMC7354531 32604961

[CR151] Kovač Tomas M, Jurčević Šangut I (2025) New insights into mycotoxin contamination, detection, and mitigation in food and feed systems. Toxins 17(10):515 10.3390/toxins17100515 PMC12567597 41150215

[CR152] Kowalska K, Habrowska-Górczyńska DE, Piastowska-Ciesielska AW (2016) Zearalenone as an endocrine disruptor in humans. Environ Toxicol Pharmacol 48:141–149 10.1016/j.etap.2016.10.015 27771507

[CR153] Kpembi IA, Oswald IP (2016) Toxicological interactions between mycotoxins. In book: Alvito P, Louro H, Silva M and Vasco E. eds. Food Contaminants and Human Health: Challenges in chemical mixtures, Lisbon, Portugal

[CR154] Kumar S, Sinha A, Kumar R, Singh V, Hooda KS, Nath K (2020) Storage fungi and mycotoxins. Seed-Borne Dis Agricultural Crops: Detect Diagnosis Manage, 821–861

[CR155] Kumar N, Upadhyay G, Chhetri KB, Harsha B, Malik GK, Kumar R, Jasrotia P, Samota SR, Kumar N, Chhokar R (2023) Pre-and post-harvest management of wheat for improving the productivity, quality, and resource utilization efficiency. Wheat science. CRC Press, pp 57–106

[CR158] Kyei NN, Cramer B, Humpf HU, Degen GH, Ali N, Gabrysch S (2022) Assessment of multiple mycotoxin exposure and its association with food consumption: a human biomonitoring study in a pregnant cohort in rural Bangladesh. Arch Toxicol 96(7):2123–2138 10.1007/s00204-022-03288-0 PMC9151532 35441239

[CR159] Lalpekhlua K, Tirkey A, Saranya S, Babu PJ (2024) Post-harvest management strategies for quality preservation in crops. Int J Veg Sci 30(5):587–635

[CR161] Leung MC, Smith TK, Karrow NA, Boermans HJ (2007) Effects of foodborne *Fusarium* mycotoxins with and without a polymeric glucomannan mycotoxin adsorbent on food intake and nutrient digestibility, body weight, and physical and clinicopathologic variables of mature dogs. Am J Vet Res 68(10):1122–1129 10.2460/ajvr.68.10.1122 17916021

[CR163] Li W, Hongyu Z, Ruixue Z, Yang W, Yijin WC, Yao H, Rong J, R. and, Shiqiang J (2021b) Fumonisin B_1_ exposure adversely affects porcine oocyte maturation in vitro by inducing mitochondrial dysfunction and oxidative stress. Theriogenology 164, 1–11 10.1016/j.theriogenology.2021.01.011 33529806

[CR162] Li L, Zhang T, Ren X, Li B, Wang S (2021a) Male reproductive toxicity of zearalenone—meta-analysis with mechanism review. Ecotoxicol Environ Saf 221:112457 10.1016/j.ecoenv.2021.112457 34175827

[CR164] Liang Z, Ren Z, Gao S, Chen Y, Yang Y, Yang D, Deng J, Zuo Z, Wang Y, Shen L (2015) Individual and combined effects of deoxynivalenol and zearalenone on mouse kidney. Environ Toxicol Pharmacol 40(3):686–91 10.1016/j.etap.2015.08.029 26407231

[CR165] Liao X, Li Y, Long N, Xu Q, Li P, Wang J, Zhou L, Kong W (2023) Multi-mycotoxin detection and human exposure risk assessment in medicinal foods. Food Res Int 164:112456 10.1016/j.foodres.2023.112456 36738010

[CR166] Ling A, Guo J, Guo W, Yang J, Zhao Z (2019) Effects of zearalenone on laying performance, hematological indices and reproductive hormone levels in laying hens. Acta Agriculturae Shanghai 35(4):100–106

[CR167] Ling A, Sun L, Guo W, Sun S, Yang J, Zhao Z (2020) Individual and combined cytotoxic effects of T-2 toxin and its four metabolites on porcine Leydig cells. Food Chem Toxicol 139:111277 10.1016/j.fct.2020.111277 32217092

[CR168] Liu J, Applegate T (2020) Zearalenone (ZEN) in livestock and poultry: dose, toxicokinetics, toxicity and estrogenicity. Toxins 12(6):377 10.3390/toxins12060377 PMC7354539 32517357

[CR169] Liu M, Gao R, Meng Q, Zhang Y, Bi C, Shan A (2014) Toxic effects of maternal zearalenone exposure on intestinal oxidative stress, barrier function, immunological and morphological changes in rats. PLoS One 9(9):e106412 10.1371/journal.pone.0106412 PMC4152245 25180673

[CR170] Liu DW, Liu HY, Zhang H, Cao MC, Sun Y, Wu WD, Lu CH (2016) Potential natural exposure of endangered red-crowned crane (Grus japonensis) to mycotoxins aflatoxin B1, deoxynivalenol, zearalenone, T-2 toxin, and ochratoxin A. J Zhejiang Univ - Sci B 17:158–168 10.1631/jzus.B1500211 PMC4757585 26834016

[CR171] Liu X, Fan L, Yin S, Chen H, Hu H (2019) Molecular mechanisms of fumonisin B_1_-induced toxicities and its applications in the mechanism-based interventions. Toxicon 167:1–5 10.1016/j.toxicon.2019.06.009 31173793

[CR172] Liu WC, Pushparaj K, Meyyazhagan A, Arumugam VA, Pappuswamy M, Bhotla HK, Baskaran R, Issara U, Balasubramanian B, Khaneghah AM (2022) Ochratoxin A as an alarming health threat for livestock and human: A review on molecular interactions, mechanism of toxicity, detection, detoxification, and dietary prophylaxis. Toxicon 213:59–75 10.1016/j.toxicon.2022.04.012 35452686

[CR173] Llorens Castelló P, Sacco MA, Aquila I, Moltó Cortés JC, Juan García C (2022) Evaluation of zearalenones and their metabolites in chicken, pig and lamb liver samples. Toxins 14(11):782 10.3390/toxins14110782 PMC9692590 36422956

[CR174] Lumsangkul C, Chiang H-I, Lo N-W, Fan Y-K, Ju J-C (2019) Developmental toxicity of mycotoxin fumonisin b1 in animal embryogenesis: An Overview. Toxins 11:114 10.3390/toxins11020114 PMC6410136 30781891

[CR175] Lumsangkul C, Tso KH, Fan YK, Chiang HI, Ju JC (2021) Mycotoxin fumonisin B_1_ interferes sphingolipid metabolisms and neural tube closure during early embryogenesis in brown tsaiya ducks. Toxins 13(11):743 10.3390/toxins13110743 PMC8619080 34822527

[CR177] Luongo D, Severino L, Bergamo P, De Luna R, Lucisano A, Rossi M (2006) Interactive effects of fumonisin B1 and alpha-zearalenol on proliferation and cytokine expression in Jurkat Tcells. Toxicol Vitro 20:1403–1410 10.1016/j.tiv.2006.06.006 16899350

[CR178] Mahato DK, Pandhi S, Kamle M, Gupta A, Sharma B, Panda BK, Srivastava S, Kumar M, Selvakumar R, Pandey AK, Suthar P (2022) Trichothecenes in food and feed: occurrence, impact on human health and their detection and management strategies. Toxicon 208:62–77 10.1016/j.toxicon.2022.01.011 35104534

[CR179] Malir F, Louda M, Toman J, Ostry V, Pickova D, Pacovsky J, Brodak M, Pfohl-Leszkowicz A (2021) Investigation of ochratoxin A biomarker in biological materials obtained from patients suffering from renal cell carcinoma. Food Chem Toxicol 158:112669 10.1016/j.fct.2021.112669 34774926

[CR180] Mandal P, Lanaridi O, Warth B, Ansari KM (2024) Metabolomics as an emerging approach for deciphering the biological impact and toxicity of food contaminants: the case of mycotoxins. Crit Rev Food Sci Nutr 64(27):9859–9883 10.1080/10408398.2023.2217451 37283072

[CR181] Marasas WF, Riley RT, Hendricks KA, Stevens VL, Sadler TW, Gelineau-van Waes J, Missmer SA, Cabrera J, Torres O, Gelderblom WC, Allegood J (2004) Fumonisins disrupt sphingolipid metabolism, folate transport, and neural tube development in embryo culture and in vivo: a potential risk factor for human neural tube defects among populations consuming fumonisin-contaminated maize. J Nutr 134(4):711–716 10.1093/jn/134.4.711 15051815

[CR182] Marcelloni AM, Pigini D, Chiominto A, Gioffrè A, Paba E (2024) Exposure to airborne mycotoxins: the riskiest working environments and tasks. Annals Work Exposures Health 68(1):19–35 10.1093/annweh/wxad070 PMC10773202 38016180

[CR316] Maris E, Ndlangamandla P, Adelusi OA, Akinmoladun OF, Odukoya JO, Fagbohun RT, Oyeyinka SA, Sekhejane P, Pero-Gascon R, De Boevre M, Croubels S. Nixtamalization of Maize to Reduce Mycotoxin Exposure: A Human Biomonitoring Intervention Study in Soweto, South Africa. Toxins. 2025 Oct 26;17(11):527. 10.3390/toxins17110527 PMC12656582 41295842

[CR183] Massarolo KC, Kupski L, Cerqueira MBR, Buffon JG, Badiale-Furlong E (2025) Bioaccessibility of Aflatoxins in Grains and Flours. Bioaccessibility of Compounds in Foods and Byproducts. Springer US, New York, NY, pp 79–91

[CR184] Matchado A, Smith JW, Schulze KJ, Groopman JD, Kortekangas E, Chaima D, Arnold CD, Maleta K, Ashorn U, Ashorn P, Dewey KG (2023) Child aflatoxin exposure is associated with poor child growth outcomes: a prospective cohort study in rural Malawi. Curr Dev Nutr 7(7):101962 10.1016/j.cdnut.2023.101962 PMC10328803 37426291

[CR185] McNutt SH, Purwin P, Murray C (1928) Vulvovaginitis in swine. J Am Vet Med Assoc 73:484–492

[CR186] Medina Á, González-Jartín JM, Sainz MJ (2017) Impact of global warming on mycotoxins. Curr Opin Food Sci 18:76–81

[CR187] Meneely JP, Kolawole O, Haughey SA, Miller SJ, Krska R, Elliott CT (2023) The challenge of global aflatoxins legislation with a focus on peanuts and peanut products: a systematic review. Exposure Health 15(2):467–487

[CR188] Mihalcea A, Amariei S (2022) Study on Contamination with Some Mycotoxins in Maize and Maize-Derived Foods. Appl Sci 12(5):2579

[CR190] Misihairabgwi JM, Ezekiel CN, Sulyok M, Shephard GS, Krska R (2017) Mycotoxin contamination of foods in Southern Africa: A 10-year review (2007–2016). Crit Rev Food Sci Nutr 59(1):43–58 10.1080/10408398.2017.1357003 28799776

[CR191] Missmer SA, Suarez L, Felkner M, Wang E, Merrill AH Jr, Rothman KJ, Hendricks KA (2006) Exposure to fumonisins and the occurrence of neural tube defects along the Texas–Mexico border. Environ Health Perspect 114(2):237–241 10.1289/ehp.8221 PMC1367837 16451860

[CR192] Mngqawa P, Shephard GS, Green IR, Ngobeni SH, De Rijk TC, Katerere DR (2016) Mycotoxin contamination of home-grown maize in rural northern South Africa (Limpopo and Mpumalanga Provinces). Food Addit Contaminants: Part B 9(1):38–45 10.1080/19393210.2015.1121928 26600208

[CR193] Mobio TA, Tavan E, Baudrimont I, Anane R, Carratú M-R, Sanni A, Gbeassor MF, Shier TW, Narbonne J-F, Creppy EE (2003) Comparative study of the toxic effects of fumonisin B_1_ in rat C6 glioma cells and p53-null mouse embryo fibroblasts. Toxicology 183(1–3):65–75 10.1016/s0300-483x(02)00441-9 12504343

[CR194] Mokubedi SM, Phoku JZ, Changwa RN, Gbashi S, Njobeh PB (2019) Analysis of mycotoxins contamination in poultry feeds manufactured in selected provinces of south Africa using UHPLC-MS/MS. Toxins 11(8):452 10.3390/toxins11080452 PMC6722855 31382387

[CR195] Monteiro CS, Faria MA, Pinto E, Cunha SC (2026) Age-dependent bioaccessibility of citrinin, ochratoxin A, and zearalenone in boiled white rice: a comparative INFOGEST in vitro study. Food Res Int. 10.1016/j.foodres.2026.119866 42632655

[CR197] Muñoz-Solano B, González-Peñas E (2023) Co-occurrence of mycotoxins in feed for cattle, pigs, poultry, and sheep in Navarra, a region of northern Spain. Toxins 15(3):172 10.3390/toxins15030172 PMC10057204 36977063

[CR196] Munkvold GP, Arias S, Taschl I, Gruber-Dorninger C (2019) Mycotoxins in corn: Occurrence, impacts, and management. Corn. AACC International Press, pp 235–287

[CR198] Mutiga SK, Hoffmann V, Harvey J, Milgroom MG, Nelson R (2015) Approximately 25% of the world’s food crop are affected each year by mycotoxin. Phytopathology, 105

[CR199] Mwihia EW, Mbuthia PG, Eriksen GS, Gathumbi JK, Maina JG, Mutoloki S, Waruiru RM, Mulei IR, Lyche JL (2018) Occurrence and levels of aflatoxins in fish feeds and their potential effects on fish in Nyeri, Kenya. Toxins 10(12):543 10.3390/toxins10120543 PMC6315670 30562952

[CR200] Nabwire WR, Ombaka J, Dick CP, Strickland C, Tang L, Xue KS, Wang JS (2020) Aflatoxin in household maize for human consumption in Kenya, East Africa. Food Addit Contaminants: Part B 13(1):45–51 10.1080/19393210.2019.1690053 31775581

[CR201] Nada S, Nikola T, Bozidar U, Ilija D, Andreja R (2022) Prevention and practical strategies to control mycotoxins in the wheat and maize chain. Food Control 136:108855

[CR202] Nayakwadi S, Ramu R, Kumar SA, Kumar GV, Rajukumar K, Kumar V, Shirahatti PS, Rashmi L, Basalingappa KM (2020) Toxico-pathological studies on the effects of T-2 mycotoxin and their interaction in juvenile goats. PLoS ONE 15:0229463 10.1371/journal.pone.0229463 PMC7098593 32214355

[CR203] Nazari L, Pattori E, Manstretta V, Terzi V, Morcia C, Somma S, Moretti A, Ritieni A, Rossi V (2018) Effect of temperature on growth, wheat head infection, and nivalenol production by *Fusarium poae*. Food Microbiol 76:83–90 10.1016/j.fm.2018.04.015 30166194

[CR204] Nešić K, Habschied K, Mastanjević K (2021) Possibilities for the biological control of mycotoxins in food and feed. Toxins 13(3):198 10.3390/toxins13030198 PMC8001018 33801997

[CR205] Niaz K, Shah SZA, Khan F, Bule M (2020) Ochratoxin A–induced genotoxic and epigenetic mechanisms lead to Alzheimer disease: its modulation with strategies. Environ Sci Pollut Res 27(36):44673–44700 10.1007/s11356-020-08991-y 32424756

[CR206] Nie T, Li J, You L, Wu Q (2025) Environmental mycotoxins: A potential etiological factor for neurodegenerative diseases? Toxicology 511:154056 10.1016/j.tox.2025.154056 39814257

[CR207] Nji QN, Babalola OO, Ekwomadu TI, Nleya N, Mwanza M (2022) Six main contributing factors to high levels of mycotoxin contamination in African foods. Toxins (Basel) 14(5):318 10.3390/toxins14050318 PMC9146326 35622564

[CR208] Njoroge S, Matumba L, Kanenga K, Siambi M, Waliyar F, Maruwo J, Machinjiri N, Monyo ES (2017) Aflatoxin B_1_ levels in groundnut products from local markets in Zambia. Mycotoxin Res 33(2):113–119 10.1007/s12550-017-0270-5 28124218

[CR209] Obremski K, Gajecki M, Zwierzohowski W, Bakula T, Apoznanski J, Wojciechowski J (2003) The level of zearalenone and alpha-zearalenol in the blood of gilts with clinical symptoms of toxicosis, fed diets with a low zearalenone content. J Anim Feed Sci 12(3):529–538

[CR210] Odukoya JO, De Saeger S, De Boevre M, Adegoke GO, Audenaert K, Croubels S, Antonissen G, Vermeulen K, Gbashi S, Njobeh PB (2021) Effect of selected cooking ingredients for nixtamalization on the reduction of *Fusarium* mycotoxins in maize and sorghum. Toxins 13(1):27 10.3390/toxins13010027 PMC7823315 33406676

[CR211] Odukoya JO, De Saeger S, De Boevre M, Adegoke GO, Devlieghere F, Croubels S, Antonissen G, Njobeh PB (2024) Influence of traditional dehulling on mycotoxin reduction and GC-HRTOF-MS metabolites profile of fermented maize products. Heliyon. 10.1016/j.heliyon.2023.e23025 PMC10776939 38205294

[CR212] Okechukwu VO, Adelusi OA, Kappo AP, Njobeh PB, Mamo MA (2023) Aflatoxins: occurrence, biosynthesis, mechanism of action and effects, conventional/emerging detection techniques. Food Chem 436:137775 10.1016/j.foodchem.2023.137775 37866099

[CR213] Okori F, Cherotich S, Baidhe E, Komakech AJ, Banadda N (2022) Grain hermetic storage and post-harvest loss reduction in sub-saharan Africa: Effects on grain damage, weight loss, germination, insect infestation, and mold and mycotoxin contamination. J Biosystems Eng 47(1):48–68

[CR214] Okoth SA, Ohingo M (2004) Dietary aflatoxin exposure and impaired growth in young children from Kisumu District, Kenya: Cross sectional study. Afr J Health Sci 11(1):43–54 17298116

[CR216] Omotayo OP, Omotayo AO, Mwanza M, Babalola OO (2019b) Prevalence of mycotoxins and their consequences on human health. Toxicol Res 35(1):1–7 10.5487/TR.2019.35.1.001 PMC6354945 30766652

[CR215] Omotayo OP, Omotayo AO, Babalola OO, Mwanza M (2019a) Comparative study of aflatoxin contamination of winter and summer ginger from the Northwest Province of South Africa. Toxicol Rep 6:489–495 10.1016/j.toxrep.2019.05.011 PMC6554596 31194138

[CR217] Pack ED, Weiland S, Musser R, Schmale DG (2021) Survey of zearalenone and type-B trichothecene mycotoxins in swine feed in the USA. Mycotoxin Res 37:297–313 10.1007/s12550-021-00442-y 34537950

[CR218] Pavlov I, Payros D, Oswald IP, Alassane-Kpembi I (2025) The mycotoxin interactions in the frame of the chemical exposome: from the toxicodynamics to the pharmacokinetic outcomes. Mycotoxins. Wageningen Academic, pp 53–70

[CR219] Peivasteh-Roudsari L, Pirhadi M, Shahbazi R, Eghbaljoo-Gharehgheshlaghi H, Sepahi M, Mirza Alizadeh A, Tajdar-oranj B, Jazaeri S (2022) Mycotoxins: Impact on health and strategies for prevention and detoxification in the food chain. Food Reviews Int 38:193–224

[CR220] Perrone G, Ferrara M, Medina A, Pascale M, Magan N (2020) Toxigenic fungi and mycotoxins in a climate change scenario: Ecology, genomics, distribution, prediction and prevention of the risk. Microorganisms 8(10):1496 10.3390/microorganisms8101496 PMC7601308 33003323

[CR221] Pestka JJ (2010) Deoxynivalenol: mechanisms of action, human exposure, and toxicological relevance. Arch Toxicol 84:663–679 10.1007/s00204-010-0579-8 20798930

[CR223] Phan LTK, De Saeger S, Eeckhout M, Jacxsens L (2023) Public health risk due to aflatoxin and fumonisin contamination in rice in the Mekong Delta, Vietnam. Int J Food Contam 10(1):4

[CR224] Pinhão M, Tavares AM, Loureiro S, Louro H, Alvito P, Silva MJ (2020) Combined cytotoxic and genotoxic effects of ochratoxin A and fumonisin B1 in human kidney and liver cell models. Toxicol In Vitro 68:104949 10.1016/j.tiv.2020.104949 32717212

[CR225] Pitt JI, Miller JD (2017) A concise history of mycotoxin research. J Agric Food Chem 65(33):7021–7033 10.1021/acs.jafc.6b04494 27960261

[CR226] Platzer A, Cherkaoui Y, Novak B, Schatzmayr G (2025) Investigating the Correlations Between Weather Factors and Mycotoxin Contamination in Corn: Evidence from Long-Term Data. Toxins 17(2):77 10.3390/toxins17020077 PMC11861693 39998094

[CR227] Pleadin J, Frece J, Lešić T, Zadravec M, Vahčić N, Staver M, M. and, Markov K (2017) Deoxynivalenol and zearalenone in unprocessed cereals and soybean from different cultivation regions in Croatia. Food Addit Contaminants: Part B 10(4):268–274 10.1080/19393210.2017.1345991 28635371

[CR228] Poapolathep S, Dzuman Z, Pongpraket M, Khidkhan K, Klangkaew N, Phaochoosak N, Zhang Z, Hajslova J, Poapolathep A (2026) Simultaneous determination and occurrence assessment of 55 mycotoxins in tea and spices using liquid chromatography-high resolution mass spectrometry. Mycotoxin Res 42(1):7 10.1007/s12550-025-00624-y 41317257

[CR229] Pongpraket M, Poapolathep A, Wongpanit K, Tanhan P, Giorgi M, Zhang Z, Li P, Poapolathep S (2020) Exposure assessment of multiple mycotoxins in black and white sesame seeds consumed in Thailand. J Food Prot 83(7):1198–1207 10.4315/JFP-19-597 32577757

[CR230] Prakasham K, Gurrani S, Shiea J, Wu MT, Wu CF, Lin YC, Tsai B, Huang PC, Andaluri G, Ponnusamy VK (2023) Ultra-sensitive determination of ochratoxin A in coffee and tea samples using a novel semi-automated in-syringe based coagulant-assisted fast mycotoxin extraction (FaMEx) technique coupled with UHPLC-MS/MS. Food Chem 417:135951 10.1016/j.foodchem.2023.135951 36934712

[CR231] Prakoso YA (2019) Recent update: effects of aflatoxin in broiler chickens. J World Poult Res 9(2):68–77

[CR232] Proctor RH, McCormick SP, Gutiérrez S (2020) Genetic bases for variation in structure and biological activity of trichothecene toxins produced by diverse fungi. Appl Microbiol Biotechnol 104:5185–5199 10.1007/s00253-020-10612-0 32328680

[CR233] Puvača N, Pelić Ljubojević, D. and, Tufarelli V (2023) Mycotoxins Adsorbents in Food Animal Production

[CR234] Qing H, Huang S, Zhan K, Zhao L, Zhang J, Ji C, Ma Q (2022) Combined toxicity evaluation of ochratoxin A and aflatoxin B1 on kidney and liver injury, immune inflammation, and gut microbiota alteration through a pair-feeding pullet model. Front Immunol 13:920147. 10.3389/fimmu.2022.920147 PMC9373725 35967406

[CR235] Ráduly Z, Szabó A, Mézes M, Balatoni I, Price RG, Dockrell ME, Pócsi I, Csernoch L (2023) New perspectives in application of kidney biomarkers in mycotoxin-induced nephrotoxicity, with a particular focus on domestic pigs. Front Microbiol 14:1085818 10.3389/fmicb.2023.1085818 PMC10140568 37125184

[CR236] Rai A, Das M, Tripathi A (2020) Occurrence and toxicity of a fusarium mycotoxin, zearalenone. Crit Rev Food Sci Nutr 60(16):2710–2729 10.1080/10408398.2019.1655388 31446772

[CR237] Rajaura S, Chauhan P, Chandra H, Bhardwaj N (2023) Aflatoxin B_1_ administration induces reactive oxygen species production and apoptosis of erythrocytes in mice. Toxicon 221:106963 10.1016/j.toxicon.2022.106963 36356707

[CR238] Ramos H, Araújo AM, Ferreira IM, Faria MA (2025) The neurotoxic impact of food chemical contaminants: a growing concern? Curr Opin Food Sci. 10.1016/j.cofs.2025.101369

[CR239] Rehman N, Shakeel M, Mohammad B, Shakir AH, Khan D (2022) Mycotoxins in Dairy Feed and its Harmful Impact on Animal Health: Diagnostic Aids and Treatment: A Big Animal Health Challenge. Chem Pharm 2(1):1–9

[CR240] Reisinger N, Schürer-Waldheim S, Mayer E, Debevere S, Antonissen G, Sulyok M, Nagl V (2019) Mycotoxin occurrence in maize silage—A neglected risk for bovine gut health? Toxins 11(10):577 10.3390/toxins11100577 PMC6832361 31590302

[CR241] Ren Z, Deng H, Deng Y, Liang Z, Deng J, Zuo Z, Hu Y, Shen L, Yu S, Cao S (2017) Combined effects of deoxynivalenol and zearalenone on oxidative injury and apoptosis in porcine splenic lymphocytes in vitro. Exp Toxicol Pathol 69(8):612–617 10.1016/j.etp.2017.05.008 28587827

[CR242] Renn O, Laubichler M, Lucas K, Kröger W, Schanze J, Scholz RW, Schweizer PJ (2022) Systemic risks from different perspectives. Risk Anal 42:1902–1920 10.1111/risa.13657 33331037

[CR243] Rheeder JP, Marasas WFO, Vismer HF (2002) Production of fumonisin analogs by *Fusarium* Species. Appl Environ Microbiol 68(5):2101–2105 10.1128/AEM.68.5.2101-2105.2002 PMC127586 11976077

[CR245] Rokvić N, Aksentijević K, Kureljušić J, Vasiljević M, Todorović N, Zdravković N, Stojanac N (2020) Occurrence and transfer of mycotoxins from ingredients to fish feed and fish meat of common carp (Cyprinus carpio) in Serbia. World Mycotoxin J 13(4):545–552

[CR246] Rong X, Wang Y, Ouyang F, Song W, Li S, Li F, Zhao S, Li D (2023) Combined effects of zearalenone and deoxynivalenol on oxidative stress, hepatotoxicity, apoptosis, and inflammation in zebrafish embryos. Sci Total Environ 859:160233 10.1016/j.scitotenv.2022.160233 36403834

[CR247] Ruan ML, Wang J, Xia ZY, Li XW, Zhang B, Wang GL, Wu Y-Y, Han Y, Deng J, Sun LH (2023) An integrated mycotoxin-mitigating agent can effectively mitigate the combined toxicity of AFB1, DON and OTA on the production performance, liver and oviduct health in broiler breeder hens. Food Chem Toxicol 182:114159 10.1016/j.fct.2023.114159 37913901

[CR248] Ruiz M-J, Macáková P, Juan-García A, Font G (2011a) Cytotoxic effects of mycotoxin combinations in mammalian kidney cells. Food Chem Toxicol 49:2718–2724 10.1016/j.fct.2011.07.021 21798303

[CR249] Ruiz MJ, Franzova P, Juan-García A, Font G (2011b) Toxicological interactions between the mycotoxins beauvericin, deoxynivalenol and T-2 toxin in CHO-K1 cells in vitro. Toxicon 58:315–326 10.1016/j.toxicon.2011.07.015 21821061

[CR250] Salah-Abbès JB, Abbès S, Abdel-Wahhab MA, Oueslati R (2009) Raphanus sativus extract protects against zearalenone induced reproductive toxicity, oxidative stress and mutagenic alterations in male Balb/c mice. Toxicon 53:525–553 10.1016/j.toxicon.2009.01.013 19673099

[CR251] Schoevers EJ, Santos RR, Colenbrander B, Fink-Gremmels J, Roelen BA (2012) Transgenerational toxicity of zearalenone in pigs. Reprod Toxicol 34(1):110–119 10.1016/j.reprotox.2012.03.004 22484360

[CR252] Sheng K, Zhang H, Yue J, Gu W, Gu C, Zhang H, Wu W (2018) Anorectic response to the trichothecene T-2 toxin correspond to plasma elevations of the satiety hormone glucose-dependent insulinotropic polypeptide and peptide YY_3–36_. Toxicology 402:28–36 10.1016/j.tox.2018.04.007 29689362

[CR253] Silva LJ, Teixeira AC, Pereira AM, Pena A, Lino CM (2020) Ochratoxin A in beers marketed in Portugal: occurrence and human risk assessment. Toxins 12(4):249 10.3390/toxins12040249 PMC7232135 32290581

[CR254] Smith MC, Madec S, Coton E, Hymery N (2016) Natural co-occurrence of mycotoxins in foods and feeds and their in vitro combined toxicological effects. Toxins 8(4):94 10.3390/toxins8040094 PMC4848621 27023609

[CR255] Soriano JM, Dragacci S (2004) Occurrence of fumonisins in foods. Food Res Int 37:985–1000

[CR256] Stanciu O, Juan C, Berrada H, Miere D, Loghin F, Mañes J (2019) Trichothecene and zearalenone presence in Romanian wheat relative to weather conditions. Toxins 11(3):163 10.3390/toxins11030163 PMC6468749 30875933

[CR257] Stoev SD (2015) Foodborne mycotoxicoses, risk assessment and underestimated hazard of masked mycotoxins and joint mycotoxin effects or interaction. Environ Toxicol Pharmacol 39(2):794–809 10.1016/j.etap.2015.01.022 25734690

[CR258] Stoev SD (2017) Balkan Endemic Nephropathy–Still continuing enigma, risk assessment and underestimated hazard of joint mycotoxin exposure of animals or humans. Chemico-Biol Interact 261:63–79 10.1016/j.cbi.2016.11.018 27871899

[CR259] Stoev SD (2022) Studies on teratogenic effect of ochratoxin A given via mouldy diet in mice in various sensitive periods of the pregnancy and the putative protection of phenylalanine. Toxicon 210:32–38 10.1016/j.toxicon.2022.02.012 35189180

[CR260] Streit E, Naehrer K, Rodrigues I, Schatzmayr G (2013) Mycotoxin occurrence in feed and feed raw materials worldwide: long-term analysis with special focus on Europe and Asia. J Sci Food Agric 93:2892–2899 10.1002/jsfa.6225 23670211

[CR261] Su Y, Sun Y, Ju D, Chang S, Shi B, Shan A (2018) The detoxification effect of vitamin C on zearalenone toxicity in piglets. Ecotoxicol Environ Saf 158:284–292 10.1016/j.ecoenv.2018.04.046 29715633

[CR262] Sulzberger S, Melnichenko S, Cardoso F (2017) Effects of clay after an aflatoxin challenge on aflatoxin clearance, milk production, and metabolism of Holstein cows. J Dairy Sci 100:1856–1869 10.3168/jds.2016-11612 28088422

[CR263] Sun LH, Lei MY, Zhang NY, Gao X, Li C, Krumm CS, Qi DS (2015) Individual and combined cytotoxic effects of aflatoxin B1, zearalenone, deoxynivalenol and fumonisin B1 on BRL 3A rat liver cells. Toxicon 95:6–12 10.1016/j.toxicon.2014.12.010 25549941

[CR264] Sun T, Ouyang H, Sun P, Zhang W, Wang Y, Cheng S, Chen G (2022) Postharvest UV-C irradiation inhibits blackhead disease by inducing disease resistance and reducing mycotoxin production in ‘Korla’fragrant pear (Pyrus sinkiangensis). Int J Food Microbiol 362:109485 10.1016/j.ijfoodmicro.2021.109485 34823080

[CR265] Sun Y, Song Y, Long M, Yang S (2023) Immunotoxicity of three environmental mycotoxins and their risks of increasing pathogen infections. Toxins 15(3):187 10.3390/toxins15030187 PMC10054902 36977078

[CR266] Tadele F, Demissie B, Amsalu A, Demelash H, Mengist Z, Ambelu A, Yenew C (2023) Aflatoxin contamination of animal feeds and its predictors among dairy farms in Northwest Ethiopia: one health approach implications. Front Vet Sci 10:1123573 10.3389/fvets.2023.1123573 PMC10076730 37035821

[CR267] Tahir MA, Abbas A, Muneeb M, Bilal RM, Hussain K, Abdel-Moneim AME, Farag MR, Dhama K, Elnesr SS, Alagawany M (2022) Ochratoxicosis in poultry: occurrence, environmental factors, pathological alterations and amelioration strategies. World’s Poult Sci J 78(3):727–749

[CR269] Tardieu D, Matard-Mann M, Collén PN, Guerre P (2021) Strong alterations in the sphingolipid profile of chickens fed a dose of fumonisins considered safe. Toxins (Basel) 13(11):770 10.3390/toxins13110770 PMC8619408 34822554

[CR270] Teixido-Orries I, Molino F, Aragones-Millan A, Ramos AJ, Marín S (2025) Thermal stability of deoxynivalenol, zearalenone, and their modified forms during baking in oat biscuits. Food Control 173:111223

[CR271] Terciolo C, Bracarense AP, Souto PC, Cossalter AM, Dopavogui L, Loiseau N, Oswald IP (2019) Fumonisins at doses below EU regulatory limits induce histological alterations in piglets. Toxins (Basel) 11(9):548 10.3390/toxins11090548 PMC6784023 31546931

[CR272] Tibola CS, Fernandes JMC, Guarienti EM (2016) Effect of cleaning, sorting and milling processes in wheat mycotoxin content. Food Control 60:174–179

[CR273] Tkaczyk A, Jedziniak P, Zielonka Ł, Dąbrowski M, Ochodzki P, Rudawska A (2021) Biomarkers of deoxynivalenol, citrinin, ochratoxin A and zearalenone in pigs after exposure to naturally contaminated feed close to guidance values. Toxins (Basel) 13:750 10.3390/toxins13110750 PMC8625168 34822534

[CR274] Tominaga M, Momonaka Y, Yokose C, Tadaishi M, Shimizu M, Yamane T, Oishi Y, Kobayashi-Hattor K (2016) Anorexic action of deoxynivalenol in hypothalamus and intestine. Toxicon 118:54–60 10.1016/j.toxicon.2016.04.036 27090011

[CR275] Tripathi A, Alam A (2020) Mycotoxins, mycotoxicosis and managing mycotoxin contamination: a review. *Bio-management of postharvest diseases and mycotoxigenic fungi*, 161–180

[CR276] Umar S, Munir MT, Shah MAA, Shahzad M, Khan RA, Khan AU, Ameen K, Rafia-Munir A, Saleem F (2015) Outbreak of aflatoxicosis on a local cattle farm in Pakistan. Veterinaria 3:13–17

[CR277] Valle-Algarra FM, Eva MM, Rufino M, Jose VG, Misericordia J (2011) Determination of type A and type B trichothecenes in paprika and chili pepper using LC-triple quadrupole–MS and GC–ECD. Talanta 84(4):1112–1117 10.1016/j.talanta.2011.03.017 21530786

[CR278] Varga J, Frisvad J, Samson R (2009) A reappraisal of fungi producing aflatoxins. World Mycotoxin J 2:263–277

[CR279] Vishwakarma PK, Chander S, Nimbolkar P (2024) Efficient water management tactics for mitigating fruit crop diseases. Appl Fruit Sci 66(2):771–779

[CR280] Vlachou M, Pexara A, Solomakos N, Govaris A (2022) Ochratoxin A in slaughtered pigs and pork products. Toxins (Basel) 14(2):67 10.3390/toxins14020067 PMC8876995 35202095

[CR281] Wan LYM, Turner PC, El-Nezami H (2013) Individual and combined cytotoxic effects of Fusarium toxins (deoxynivalenol, nivalenol, zearalenone and fumonisins B1) on swine jejunal epithelial cells. Food Chem Toxicol 57:276–283 10.1016/j.fct.2013.03.034 23562706

[CR282] Wang GH, Xue CY, Chen F, Ma YL, Zhang XB, Bi YZ, Cao YC (2009) Effects of combinations of ochratoxin A and T-2 toxin on immune function of yellow-feathered broiler chickens. Poult Sci 88(3):504–510 10.3382/ps.2008-00329 19211518

[CR283] Wang Y, Xing CH, Zhang HL, Pan ZN, Sun SC (2021) Exposure to nivalenol declines mouse oocyte quality via inducing oxidative stress-related apoptosis and DNA damage. Biol Reprod 105(6):1474–1483 10.1093/biolre/ioab171 34505141

[CR284] Wang G, Li E, Gallo A, Perrone G, Varga E, Ma J, Yang B, Tai B, Xing F (2022a) Impact of environmental factors on ochratoxin A: from natural occurrence to control strategy. Environ Pollut 317:120767 10.1016/j.envpol.2022.120767 36455768

[CR285] Wang Q, Zhao Y, Chen P, Zeng R, Liang Y (2022b) Ochratoxin A and zearalenone in poultry feed samples from South China. J Food Saf 42(1):12944

[CR286] Wang X, Li S, Han Y, Hou M, Gao Z (2025) Novel strategies for efficiently detoxifying mycotoxins in plant-derived foods inspired by non-thermal technologies and biological resources: a review. Crit Rev Food Sci Nutr 65(28):5887–5913 10.1080/10408398.2024.2434723 39610154

[CR287] Wangia-Dixon RN, Nishimwe K (2020) Molecular toxicology and carcinogenesis of fumonisins: A review. J Environ Sci Health Part C 39(1):44–67 10.1080/26896583.2020.1867449 33554724

[CR288] Wattanasuntorn P, Poapolathep S, Phuektes P, Alassane-Kpembi I, Fink-Gremmels J, Oswald IP, Poapolathep A (2025) Apoptotic effect of combinations of T-2, HT-2, and diacetoxyscirpenol on human Jurkat T cells. Toxins (Basel) 17(4):203 10.3390/toxins17040203 PMC12030997 40278701

[CR290] Wegulo SN (2012) Factors influencing deoxynivalenol accumulation in small grain cereals. Toxins 4(11):1157–1180 10.3390/toxins4111157 PMC3509702 23202310

[CR291] Wei D, Wang Y, Jiang D, Feng X, Li J, Wang M (2017) Survey of *Alternaria* toxins and other mycotoxins in dried fruits in China. Toxins 9:200 10.3390/toxins9070200 PMC5535147 28672847

[CR292] Woźny M, Obremski K, Hliwa P, Gomułka P, Różyński R, Wojtacha P, Florczyk M, Segner H, Brzuzan P (2019) Feed contamination with zearalenone promotes growth but affects the immune system of rainbow trout. Fish Shellfish Immunol 84:680–694 10.1016/j.fsi.2018.10.032 30359755

[CR293] Wu W, Zhou H, He K, Pan X, Sugita-Konishi Y, Watanabe M, Zhang H, Pestka JJ (2014) Role of cholecystokinin in anorexia induction following oral exposure to the 8-ketotrichothecenes deoxynivalenol, 15-acetyldeoxynivalenol, 3-acetyldeoxynivalenol, fusarenon x, and nivalenol. Toxicol Sci 138(2):278–289 10.1093/toxsci/kft335 PMC4246666 24385417

[CR294] Wu S, Lu H, Bai Y (2019) Nrf2 in cancers: A double-edged sword. Cancer Med 8:2252–2267 10.1002/cam4.2101 PMC6536957 30929309

[CR295] Wu T, Gao J, Han B, Deng H, Han X, Xie Y, Li C, Zhan J, Huang W, You Y (2024) Determination of 10 mycotoxins in wine, baijiu, and huangjiu of the Chinese market by liquid chromatography tandem mass spectrometry and exposure estimation. Food Chem X 22:101301 10.1016/j.fochx.2024.101301 PMC10979051 38559440

[CR296] Xiong J, Xiong L, Zhou H, Liu Y, Wu L (2018) Occurrence of aflatoxin B_1_ in dairy cow feedstuff and aflatoxin M_1_ in UHT and pasteurized milk in central China. Food Control 92:386–390

[CR297] Xu F, Li Y, Cao Z, Zhang J, Huang W (2021) AFB_1_-induced mice liver injury involves mitochondrial dysfunction mediated by mitochondrial biogenesis inhibition. Ecotoxicol Environ Saf 216:112213 10.1016/j.ecoenv.2021.112213 33838459

[CR298] Xue CY, Wang GH, Chen F, Zhang XB, Bi YZ, Cao YC (2010) Immunopathological effects of ochratoxin A and T-2 toxin combination on broilers. Poult Sci 89(6):1162–1166 10.3382/ps.2009-00609 20460662

[CR299] Yan H, Sun J, Fu X, Ye J, Wang W, Cao J, Ji J, Sun X (2026) Climate Change: An Inevitable Factor in Reshaping the Contamination Level of Fungi and Mycotoxins. Compr Rev Food Sci Food Saf 25(1):e70354 10.1111/1541-4337.70354 41355604

[CR300] Yousef MS, Rezk WR, El-Naby ASAH, Mahmoud KGM, Takagi M, Miyamoto A, Megahed GA (2023) In vitro effect of zearalenone on sperm parameters, oocyte maturation and embryonic development in buffalo. Reprod Biol 23(1):100732 10.1016/j.repbio.2023.100732 36669377

[CR301] Yu S, Jia B, Liu N, Yu D, Zhang S, Wu A (2021) Fumonisin B_1_ triggers carcinogenesis via HDAC/PI3K/Akt signalling pathway in human esophageal epithelial cells. Sci Total Environ 787:147405 10.1016/j.scitotenv.2021.147405 34000555

[CR302] Zain ME (2011) Impact of mycotoxins on humans and animals. J Saudi Chem Soc 15(2):129–144

[CR303] Zaworska-Zakrzewska A, Kasprowicz-Potocka M, Twarużek M, Kosicki R, Grajewski J, Wiśniewska Z, Rutkowski A (2020) A comparison of the composition and contamination of soybean cultivated in Europe and limitation of raw soy seed content in weaned pigs’ diets. Animals : an open access journal from MDPI 10(11):1972 10.3390/ani10111972 PMC7692213 33121110

[CR304] Zebiri S, Mokrane S, Verheecke-Vaessen C, Choque E, Reghioui H, Sabaou N, Mathieu F, Riba A (2019) Occurrence of ochratoxin A in Algerian wheat and its milling derivatives. Toxin Reviews 38:206–211

[CR305] Zhang Z, Gan F, Xue H, Liu Y, Huang D, Khan AZ, Chen X, Huang K (2016) Nephropathy and hepatopathy in weaned piglets provoked by natural ochratoxin A and involved mechanisms. Exp Toxicol Pathol 68:205–213 10.1016/j.etp.2015.12.002 26702942

[CR306] Zhang J, Sheng K, Wu W, Zhang H (2018) Anorectic responses to T-2 toxin, HT-2 toxin, diacetoxyscirpenol and neosolaniol correspond to plasma elevations of neurotransmitters 5-hydroxytryptamine and substance P. Ecotoxicol Environ Saf 161:451–458 10.1016/j.ecoenv.2018.06.005 29909314

[CR307] Zhang Z, Zhang XUJ, Wang X, Xie J, Sun H, Zhang Y, Chang Q, Z. and, Liu Y (2022) Protective effect of semet on liver injury induced by ochratoxin A in rabbits. Toxins 14:628 10.3390/toxins14090628 PMC9504919 36136566

[CR308] Zhao Z, Liu N, Yang L, Deng Y, Wang J, Song S, Lin S, Wu A, Zhou Z, Hou J (2015) Multi-mycotoxin analysis of animal feed and animal-derived food using LC–MS/MS system with timed and highly selective reaction monitoring. Anal Bioanal Chem 407:7359–7368 10.1007/s00216-015-8898-5 26198112

[CR309] Zhao J, Cheng T, Xu W, Han X, Zhang J, Zhang H, Li F (2021a) Natural co-occurrence of multi-mycotoxins in unprocessed wheat grains from China. Food Control 130:108321

[CR310] Zhao L, Zhang L, Xu Z, Liu X, Chen L, Dai J, Karrow NA, Sun L (2021b) Occurrence of aflatoxin B_1_, deoxynivalenol and zearalenone in feeds in China during 2018–2020. J Anim Sci Biotechnol 12:1–12 10.1186/s40104-021-00603-0 PMC8272344 34243805

[CR311] Zhou H, George S, Li C, Gurusamy S, Sun X, Gong Z, Qian H (2017) Combined toxicity of prevalent mycotoxins studied in fish cell line and zebrafish larvae revealed that the type of interactions is dose-dependent. Aquat Toxicol 193:60–71 10.1016/j.aquatox.2017.09.030 29040830

[CR312] Zhu F, Wang Y (2022) Fumonisin B_1_ induces immunotoxicity and apoptosis of chicken splenic lymphocytes. Front Vet Sci 9:898121 10.3389/fvets.2022.898121 PMC9171430 35685341

[CR315] Zoghi A, Todorov SD, Khosravi-Darani K (2022) Potential application of probiotics in mycotoxicosis reduction in mammals and poultry. Crit Rev Toxicol 52(9):731–741 10.1080/10408444.2023.2168176 36757083

