# Supplementary material for: Mycotoxin contamination in food and feed: Occurrence, co-occurrence, combined impacts on human and animal health, climate change implications, risk assessment, and mitigation strategies
Source: Mycotoxin Res. 2026 Sep 15;42(4):70. doi: 10.1007/s12550-026-00679-5 (PMC13578126; doi:10.1007/s12550-026-00679-5)
Supplement: Supplementary file 1 [file 12550_2026_679_MOESM1_ESM.docx]

**Supplementary Table**

Supplementary Table 1: MPLs for Major Mycotoxins in food and feed in selected countries (Zinedine et al., 2009; Anukul et al., 2013; BIOMIN World Mycotoxin Survey, 2020; Kemboi et al., 2020; Chatterjee et al., 2023; European Commission, 2023)

| **Mycotoxin regulation in food across different regions and countries** | | | | | | | |
| --- | --- | --- | --- | --- | --- | --- | --- |
| Country | Food samples | AFB_1_  (μg/kg) | FB_1_  (μg/kg) | OTA  (μg/kg) | ZEN  (μg/kg) | DON  (μg/kg) | T-2 toxin  (μg/kg) |
| Europe | Dried fruits ingredients | 5 | - | 2 | - | - | - |
|  | Processed products from dried fruits | 2 | - | - | - | - | - |
|  | Dried figs | 6 | - | - | - | - | - |
|  | Unprocessed cereal grains  (wheat, millet, rice, sorghum, etc) | 5 | - | 5 | 100 | 1250 | - |
|  | Processed cereals (wheat, millet, rice, sorghum, etc.) | 2 | - | 3 | 75 | 750 | 200 |
|  | Unprocessed maize grains | 5 | 4000 | 5 | 350 | 1750 | - |
|  | Processed maize | - | 1000 | - | 100 | 750 | - |
|  | Groundnuts and other oilseeds ingredients | 8 | - | - | - | - | - |
|  | Processed products from groundnuts | 2 | - | - | - | - | - |
|  | Spices (ginger, pepper, nutmeg, turmeric, paprika, cayenne, etc.) | 5 | - | 20 | - | - | - |
|  | Bread and other bakery items | - | - | - | 50 | 500 | 25 |
|  | Baby food | 0.01 | 200 | 0.50 | 20 | 200 | - |
|  | Wine and fruit juice | - | - | 2 | - | - | - |
|  | Almonds, pistachios, apricot kernels, hazelnuts, Brazil nuts, and dried fruits | 8 | - | 10 | - | - | - |
|  | Roasted coffee beans and cocoa powder | - |  | 3 | - | - | - |
|  | Instant coffee | - | - | 5 | - | - | - |
| China | Groundnut, corn, and their products | 20 | - | - | - | - | - |
|  | Pig organs and lean pork | 0.5 | - | - | - | - | - |
|  | Cereals, grains, and fermented products | 5 | - | - | - | - | - |
|  | Soy children's products and infant formulas | 0.5 | - | - | - | - | - |
|  | All food products | - | - |  | 60 | 1,000 | - |
|  | Wine | - | - | 2 | - | - | - |
|  | Instant coffee | - | - | 10 | - | - | - |
| Malaysia | Infant food | 0.1 | - | 0.5 | - | - | - |
|  | Instant coffee | - | - | 10 | - | - | - |
| Indonesia | Corn, wheat, and their products | 15 | 2,000 | - | - | 1,750 | - |
|  | Beer | - | - | 0.2 | - | - | - |
|  | Cereal and processed raw products | - | - | 5 | - | - | - |
|  | Ready-to-eat foods | - | 1,000 | 3 | - | 500 | - |
|  | Coffee | - | - | 5 | - | - | - |
|  | Instant coffee | - | - | 10 | - | - | - |
|  | Peanut and its products | 15 | - | - | - | - | - |
| India | Cereal and Cereal Products | 15 | - | - | - | - | - |
|  | Spices | 30 | - | - | - | - | - |
|  | Nuts for further processing | 15 | - | - | - | - | - |
|  | Dried figs | 10 | - | - | - | - | - |
|  | Wheat, barley, and rye | - | - | 20 | - | - | - |
|  | Wheat | - | - | - | - | 1,000 | - |
| Singapore | All the food items | 5 | - | 2.5 | - | - | - |
|  | Bread and other bakery items | - | 100 | - | - | - | - |
|  | Infant milk and baby food | - | 25 | - | - | - | - |
| Morocco | All foods, wheat brain | 10 | - | 30 | - | - | - |
|  | Vegetable oils, cereals, wheat meal (complete) | 5 | - | - | 200 | - | - |
|  | Peanuts, pistachios, nuts, almonds, vegetable oils in pasta, and children's foods | 1 | - | - | - | - | - |
|  | Wheat meal | 3 | - | - | - | - | - |
| South Africa | All food types | 5 | - | - | - | - | - |
| Egypt | Peanuts and cereals | 5 | - | - | - | - | - |
|  | Maize | 10 | - | - | - | - | - |
|  | Coffee | - | - | 5 | - | - | - |
|  | Barley and barley flour | - | - | - | - | 1000 | - |
|  | Wheat and wheat flour | - | - | - | - | 700 | - |
| Thailand | Almonds, hazelnuts, and pistachio | 10 | - | - | - | - | - |
| Vietnam | Groundnuts, dried fruits and other oilseeds | 2 | - | 10 | - | - | - |
|  | Milk and dairy products | 5 | - |  | - | - | - |
|  | Spices | 0.1 | - | 30 | - | - | - |
|  | All cereals and their products | 2 | - | 5 | 75 | 750 | - |
|  | Infant milk and baby food | 0.1 | - | 0.5 | 20 | 200 | - |
|  | Wine and fruit juice | - | - | 2 | - | - | - |
|  | Infant noodles | - | - | - | - | 750 | - |
|  | Bread and other bakery items | - | - | - | 50 | 500 | - |
|  | Instant coffee | - | - | 10 | - | - | - |
| Philippines | Ready-to-drink food supplements | 10 | - | - | - | - | - |
|  | All cereals and cereal products | - | - | 5 | - | - | - |
|  | Instant coffee | - | - | 10 | - | - | - |
|  | All foods | - | - | 5 | - | 1,000 | - |
| South Korea | Meju | - | - | 20 | - | - | - |
|  | Infant milk and baby food | - | - | 0.5 | - | - | - |
|  | Wine and fruit juice | - | - | 2 | - | - | - |
|  | Spices | - | - | 7 | - | - | - |
|  | Instant coffee | - | - | 10 | - | - | - |
|  | Infant food | - | - | - | 20 | - | - |
|  | Cereals and grain products | - | 1,000 | - | 200 | 500 | - |
|  | Corn and corn products | - | 4,000 | - | - | 2,000 | - |
| Jordan | Almonds, pistachios, and pine nuts | 15 | - | - | - | - | - |
|  | Wheat, barley, and rye | - | - | 20 | - | 1,000 | - |
| **Mycotoxin regulations for feed and feedstuffs across different regions and countries** | | | | | | | |
| Country | Feed types | AFB_1_ | OTA | DON | ZEN | FB_1_ | T-2 toxin |
| Europe | Feedstuffs for dairy cattle and calves | 5 | - | 2,000 | 500 | 20,000 | - |
|  | Feedstuffs for fish | - | - | - | - | 10,000 | - |
|  | Feedstuffs for poultry | - | 100 | - | - | 20,000 | - |
|  | Feedstuffs for pigs | - | 250 | 900 | 100 | 5,000 | - |
| CODEX | Dairy feeds | 5 | - | - | - | - | - |
| USA | Dairy feed | - | - | - | - | 30,000 | - |
| Japan | Feed for cows (over 3 months old) | - | - | 4,000 | - | - | - |
|  | Feeds for calf, dairy cattle, pigs, and chicks | - | - | - | - | - | - |
| South Africa | Dairy feed | 5 | - | 3000 | 500 | 50,000 | - |
| Philippines | Shelled corn | 50 |  | - | - | - | - |
|  | Roasted and ground coffee | - | 5 | - | - | - | - |
|  | Soluble coffee | - | 10 | - | - | - | - |
|  | Cereals and cereal products | - | 5 | - | - | - | - |
| Indonesia | Final feed products | 50 | - | - | - | - | - |
| China | Corn products and peanut meal | - | - | - | - | - | - |
|  | All feedstuffs and grains | - | 100 | - | 500 | - | - |
|  | Corn, corn oil, and peanut oil | - | - | - | 500 | 60,000 | - |
|  | Feedstuff for calves and young lambs | - | - | 1,000 | - | 20,000 | - |
|  | Cottonseed meal, rapeseed meal, and other vegetable feedstuffs | - | - | - | - | - | - |
|  | Feedstuffs for piglets | - | - | - | 150 | - | - |
|  | Feedstuffs for young ducks, chicks, and other birds | - | - | 1,000 | 250 | 5,000 | 500 |
|  | Feedstuffs for dairy cattle | - | - | 1,000 | - | - | - |
|  | Vegetable feedstuff | - | - | 5,000 | 1,000 | - | 500 |
|  | Vegetable oil and fat | - | - | - | - | - | - |
|  | Complete feeding stuff for fishes | - | - | - | - | 10,000 | - |
| Morrocco | Complete feedstuffs for dairy animals | 5 | - | - | - | - | - |
|  | Peanuts, copra, cottonseed, babassu, maize, and their products. Complete feedstuffs for pigs and poultry | 20 | - | - | - | - | - |
|  | Complementary feedstuffs for pigs and poultry (except young animals) | 30 | - | - | - | - | - |
